# RNF144A-VRK2-G3BP1 axis regulates stress granule assembly

**DOI:** 10.1038/s41420-025-02460-6

**Published:** 2025-04-09

**Authors:** Sung Wook Kim, Jae Lee, Kyung Won Jo, Young-Hun Jeong, Won Sik Shin, Kyong-Tai Kim

**Affiliations:** 1 Department of Life Sciences, Pohang University of Science and Technology, Pohang, Gyeongbuk Republic of Korea; 2 Hesed Bio Corporation, Pohang, Gyeongbuk Republic of Korea; 3 Generative Genomics Research Center, Global Green Research & Development Center, Handong Global University, Pohang, Gyeongbuk Republic of Korea

**Keywords:** Stress signalling, Chemotherapy

## Abstract

Under the cellular stress, stress granules (SGs) help survival and proliferation of the cell. Unfortunately, the same SGs help unwanted cancer cells under stressful environment, including anti-cancer chemotherapy treatment. While SGs elevate the cancer cell’s resistance to chemotherapy, the mechanism behind the formation of SGs in cancer cell under chemotherapy treatment is still to be revealed. Here, we identified that the level of VRK2 and the phosphorylation of its novel substrate, G3BP1, are reduced when the cellular stress was increased by sodium arsenite (SA) or cisplatin treatment. We also demonstrated that the level of RNF144A is increased in response to the stress and further downregulates VRK2 through proteasomal degradation in various types of cancer cells. Furthermore, inhibition of SG formation by the overexpression of VRK2 sensitized the cells to the stress and chemotherapy. Together, our study establishes an RNF144A-VRK2-G3BP1 axis that regulates SG formation and suggest its potential usage in anti-cancer therapy.

## Introduction

Stress granules (SGs) are ribonucleoprotein granules that form in response to cellular stress [1–3]. Although the composition of SGs may vary among the types of cellular stress or context [4], the SGs tend to include stalled mRNAs, translation initiation factors, RNA-binding proteins, and non-RNA-binding proteins [5]. Traditionally, SGs are known to be responsible for the translational arrest during the stress to conserve energy from unnecessary protein synthesis [6–8]. SGs also act as the storage for mRNAs that need to be translated again once the stress is relieved [9]. More recently, SGs are found to regulate the expression of proteins under the stress as an mRNA triage [10, 11]. Through the interactions with processing bodies, SGs may prioritize certain mRNAs over the other, leading them to different fates [10, 12]. Moreover, SGs interrupt with cell death signaling and delay the cellular apoptosis. Several apoptosis-inducing factors, such as receptor of activated protein C kinase 1 (RACK1), are prevented from participating in cellular apoptosis by being recruited to SGs [13, 14]. Overall, SGs are known to facilitate the survival of the cell during stressful conditions.

Recent studies on SGs show their correlation to several diseases, such as neurodegenerative disease and cancer [15–17]. Especially, SGs are studied as the new target for anti-cancer strategy since they are highly correlated to the progression of the cancer [18–20]. The microenvironment of tumor is a source of different types of stress, such as endoplasmic reticulum stress (ER stress) and oxidative stress [21]. Furthermore, various anti-cancer chemotherapeutic agents, such as sorafenib, bortezomib, and cisplatin, also provide stress that induces SG assembly in cancer cells [22–24]. The formation of SGs in cancer cells escalates the resistance to anti-cancer chemotherapy in return [25–28]. Likewise, SGs raise numerous problems in developing the cure for the cancer, but very little is known about the mechanism behind the SG formation in cancer cells under stress.

Several studies have revealed that Ras GTPase-activating protein-binding protein 1 (G3BP1) is one of the key factors in the SG assembly [29–31]. Previous studies showed that the phosphorylation of serine 149 residue (Ser149) of G3BP1 is important in the formation of SGs [32, 33]. G3BP1 has three intrinsically disordered region (IDR) domains, and Ser149 is part of IDR1 [33, 34]. Upon the phosphorylation of Ser149, IDR1 strongly interacts with an RNA-binding domain, IDR3 of G3BP1, further inhibiting G3BP1 from participating in SG assembly [33]. However, once the phosphorylation of Ser149 is reduced by stress, IDR3 is open to interact with stalled mRNAs leading to liquid-liquid phase separation required for SG formation [33, 35]. Although recent studies have revealed that Casein Kinase 2 phosphorylates Ser149 of G3BP1 [36], a specific mechanism behind the reduction of G3BP1 phosphorylation in cancer cells under stress is still to be found. Lately, vaccinia-related kinase 2 (VRK2) and its significance in the cancer progression has been noted [37–40]. VRK2 is a serine-threonine kinase previously known for its function in cell proliferation [41, 42]. While some reports show that the level of VRK2 is upregulated in cancer, our group recently identified that the level of VRK2 decreases in response to oxidative stress [43]. Thus, there is a great possibility that the reduction in the phosphorylation of G3BP1 under the stress is mediated by a decline in VRK2 level.

Here in this study, we define a novel signaling pathway that regulates the formation of SGs in the cancer cells. We demonstrated that VRK2 phosphorylates Ser149 of G3BP1 and negatively regulates the formation of SGs. Under sodium arsenite (SA) or cisplatin-mediated stress, we found that RNF144A acts as an E3 ligase that promotes proteasomal degradation of VRK2, which induces the formation of SGs. Modulating the level of RNF144A or VRK2 regulated the formation of SGs in U2OS osteosarcoma cell line as well as its resistance to stress or anti-cancer chemotherapy. Our findings establish an RNF144A-VRK2-G3BP1 axis that regulates the SG assembly in cancer cells under stress, which could be a potential target for anti-cancer strategy.

## Results

### VRK2 directly interacts with G3BP1 and phosphorylates its Ser149 residue

**Fig. 1:**
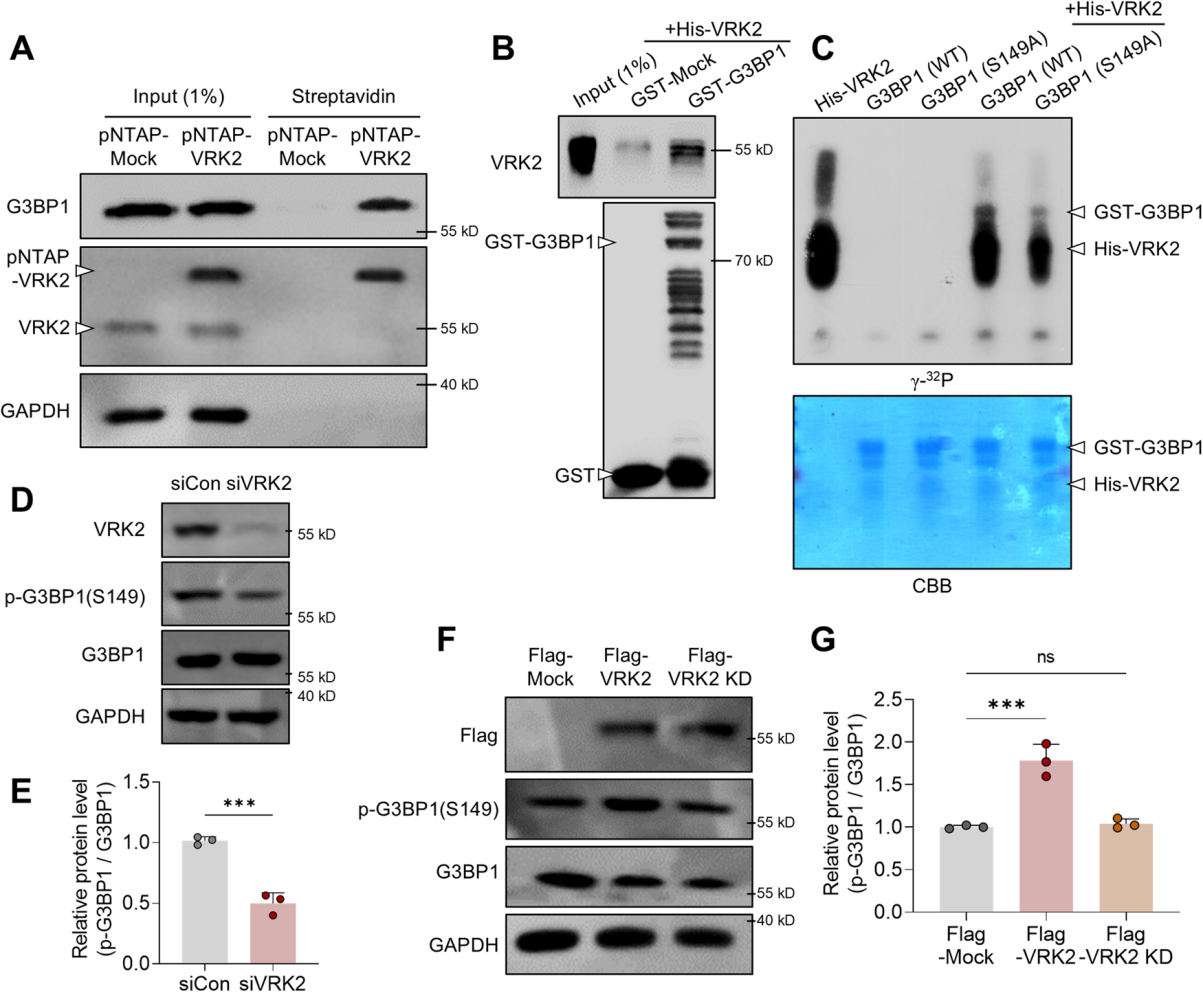
VRK2 directly interacts with G3BP1 and phosphorylates its Ser149 residue. **A** Representative immunoblot of pNTAP-streptavidin pull down assay performed with pNTAP-Mock or pNTAP-VRK2 transfected U2OS cells. **B** Representative immunoblot of GST pull down assay performed with recombinant GST-G3BP1 and His-VRK2. **C** Autoradiograph (γ-^32^P) and Coomassie brilliant blue (CBB) staining from in vitro kinase assay using recombinant GST-G3BP1 and His-VRK2; WT: wild type, S149A: mutation of Ser149 to alanine. Representative immunoblot (**D**) and quantification (**E**) of VRK2 knockdown experiments. U2OS cells were transfected with small interfering RNA for control (siCon) or for VRK2 (siVRK2). The phosphorylation of G3BP1 is normalized to total G3BP1, and GAPDH was used as loading control (*n* = 3). Representative immunoblot (**F**) and quantification (**G**) of VRK2 overexpression experiments. U2OS cells were transfected with Flag-Mock, Flag-VRK2, or Flag-VRK2 kinase dead form (VRK2 KD). The phosphorylation of G3BP1 is normalized to total G3BP1 and GAPDH was used as loading control (*n* = 3). n.s., not significant, *** *p* ≤ 0.001; unpaired Student’s t test was performed for (**E**); ordinary one-way ANOVA with Tukey’s multiple comparison test was performed for (**G**). The “n” represents the number of independent experiments. Error bars indicate SDs.

To see if VRK2 has any interactions with G3BP1, we performed immunoprecipitation (IP) using Flag-VRK2 and Flag antibody followed by mass spectrometry analysis. We identified G3BP1 as a potential binding partner of VRK2 (Supplementary Fig. 1A and B). To confirm, we proceeded with pNTAP-streptavidin pull down assay with osteosarcoma cell line, known as U2OS cells, transfected with either pNTAP-Mock or pNTAP-VRK2 (Fig. 1A and Supplemental Material). We further identified a direct interaction between VRK2 and G3BP1 through GST pull down assay (Fig. 1B and Supplemental Material). These data together demonstrate a direct interaction between VRK2 and G3BP1.

Since VRK2 is a serine/threonine protein kinase[42], we next analyzed whether VRK2 is able to phosphorylate G3BP1. Through in vitro kinase assay, we demonstrated the phosphorylation of recombinant G3BP1 by recombinant VRK2 (Fig. 1C). Since the phosphorylation of Ser149 of G3BP1 is known to be important in SG assembly [33], we used mutated form of recombinant G3BP1 (S149A) to identify the site of VRK2 phosphorylation. We identified that VRK2 phosphorylates Ser149 of G3BP1 along with other potential residues (Fig. 1C). Using cold ATP and antibody, we further confirmed the phosphorylation of Ser149 of G3BP1 by VRK2 (Supplementary Fig. 2A). To check whether VRK2 phosphorylates endogenous G3BP1, we modulated the level of VRK2 in U2OS cells through knockdown or overexpression of VRK2. While the knockdown of VRK2 reduced the phosphorylation of Ser149 of G3BP1 (Fig. 1D, E, and Supplemental Material), the overexpression of VRK2 increased the phosphorylation (Fig. 1F, G and Supplemental Material). Interestingly, when we overexpressed kinase-dead (KD) form of VRK2 in U2OS cells, the phosphorylation of G3BP1 was not increased, which indicates that the phosphorylation of G3BP1 was mediated by the kinase activity of VRK2 (Fig. 1F, G and Supplemental Material). Altogether, these evidences show that VRK2 directly interacts with G3BP1 and phosphorylates its Ser149.

### Loss of VRK2 during stress induces G3BP1-mediated SG formation

**Fig. 2:**
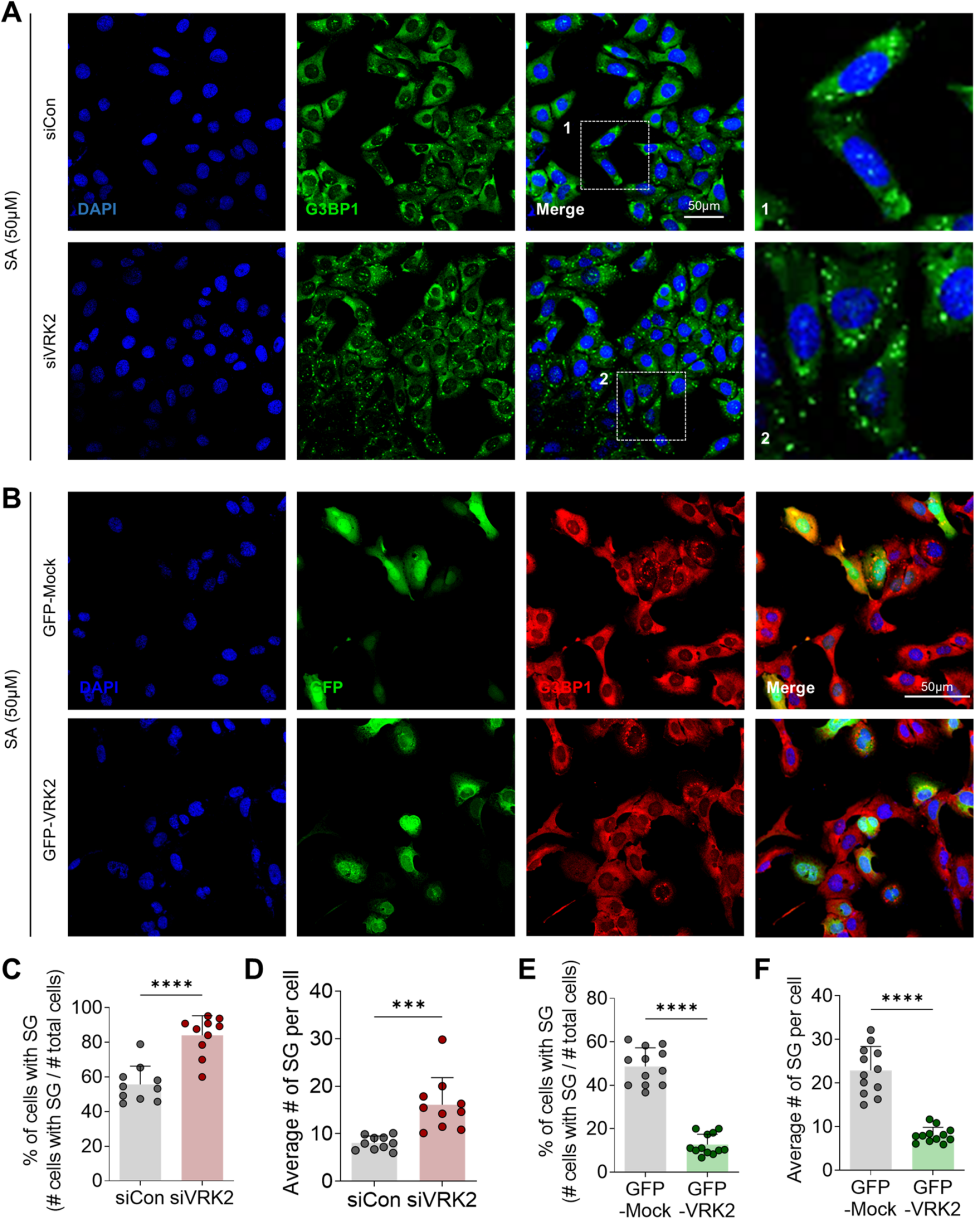
VRK2 participates in G3BP1-mediated SG formation. **A** Representative image of SG formation in VRK2-deficient U2OS cells. U2OS cells were transfected with siVRK2 and the stress was induced by the treatment with sodium arsenite (SA, 50 μM, 2 h). SGs were stained with G3BP1 (green) and the nuclei of cells were stained with DAPI (blue). Scale bar = 50 µm (**B**) Representative image of SG formation in VRK2-excessive U2OS cells. U2OS cells were transfected with GFP-VRK2 and the stress was induced by the treatment with SA (50 μM, 2 h). SGs were stained with G3BP1 (red) and the nuclei of cells were stained with DAPI (blue). Scale bar = 50 µm. The proportion of U2OS cells with SGs (**C**) and the average number of SGs per U2OS cells (**D**) were quantified in control or VRK2-deficient U2OS cells (*n* = 9). The proportion of U2OS cells with SGs (**E**) and the average number of SGs per U2OS cells (**F**) were quantified in control or VRK2-excessive U2OS cells (*n* = 12). *** *p* ≤ 0.001, **** *p* ≤ 0.0001; unpaired Student’s t test was performed for (**C**, **D**, **E**, **F**). The “n” represents the number of independent experiments. Error bars indicate SDs.

The phosphorylation of Ser149 of G3BP1 is critical in the formation of the SGs [33, 35]. Ser149 is a part of IDR1 domain of G3BP1, which strongly interacts with the IDR3 domain of G3BP1 upon phosphorylation [33]. De-phosphorylation of Ser149 of G3BP1 weakens the interaction between IDR1 and IDR3, leaving IDR3 open to interact with stalled mRNAs, further creating seed for SG formation [35]. Since we demonstrated that VRK2 phosphorylates G3BP1, we next assessed whether VRK2 participates in G3BP1-mediated SG formation. We modulated the level of VRK2 and induced the SGs by the treatment with sodium arsenite (SA), an inorganic compound that induces oxidative stress and DNA damage [44, 45]. When the level of VRK2 was knocked down, both the proportion of cells with SGs and the average number of SGs per cell were significantly increased (Fig. 2A, C, D). On the other hand, the overexpression of VRK2 by GFP-VRK2 reduced both the proportion of cells with SGs and the average number of SGs per cell (Fig. 2B, E, F). This was confirmed with U2OS cells stably expressing pNTAP-VRK2 (Supplementary Fig. 2B–D).

**Fig. 3:**
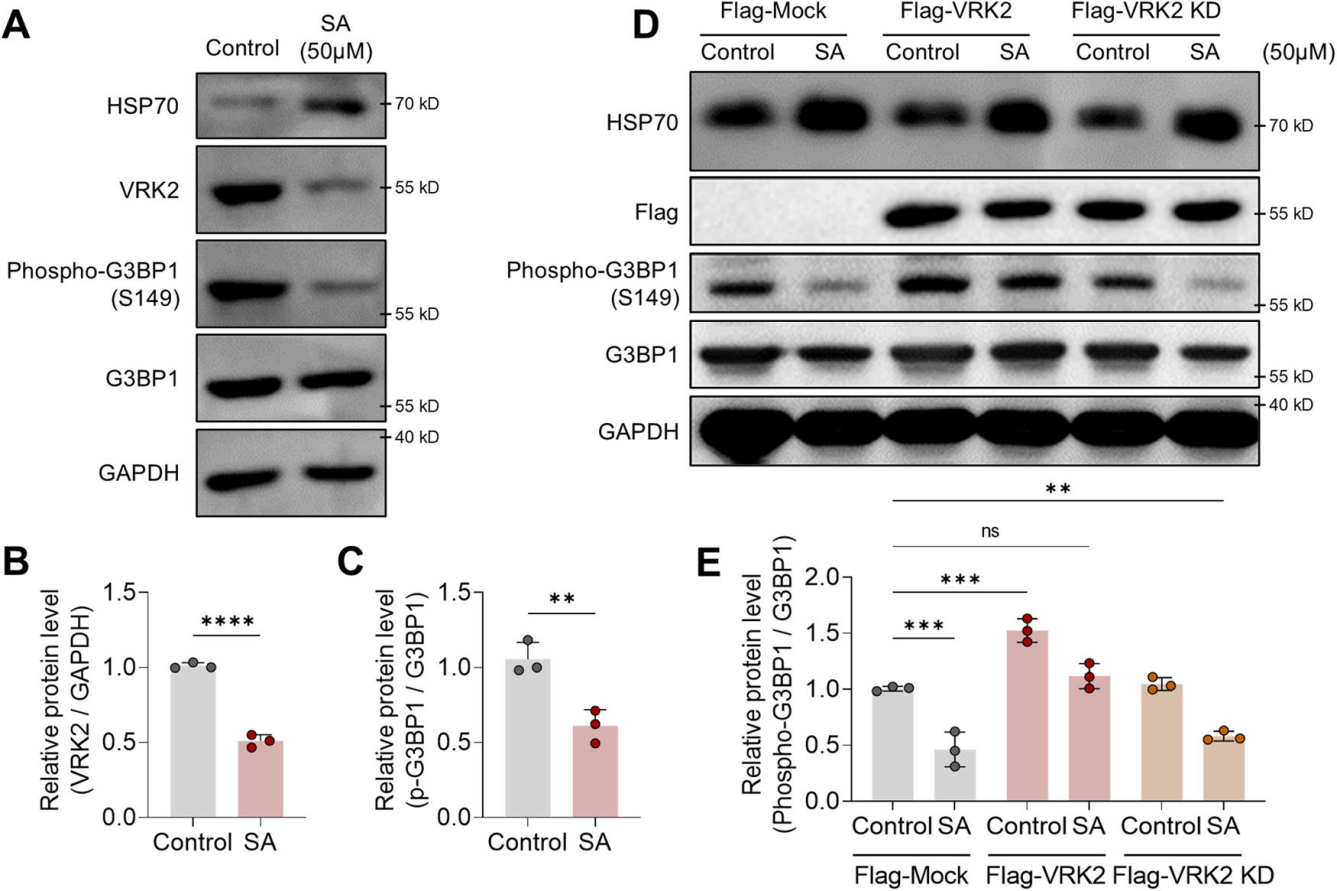
VRK2 and G3BP1 phosphorylation are downregulated during stress. Representative immunoblot (**A**) and the quantification of VRK2 (**B**) and G3BP1 phosphorylation (**C**) in stress-induced U2OS cells. The stress was induced with SA (50 μM, 8 h). HSP70 was used as the marker for SA-induced stress. The phosphorylation of G3BP1 is normalized to total G3BP1 and GAPDH was used as loading control (*n* = 3). Representative immunoblot (**D**) and the quantification of G3BP1 phosphorylation (**E**). U2OS cells were transfected with Flag-Mock, Flag-VRK2, or Flag-VRK2 KD and the stress was induced with SA (50 μM, 8 h). HSP70 was used as the marker for SA-induced stress. The phosphorylation of G3BP1 is normalized to total G3BP1 and GAPDH was used as loading control (*n* = 3). n.s., not significant, **p ≤ 0.01, ***p ≤ 0.001, ****p ≤ 0.0001; unpaired Student’s t test was performed for (**B**, **C**); ordinary one-way ANOVA with Tukey’s multiple comparison test was performed for (**E**). The “n” represents the number of independent experiments. Error bars indicate SDs.

Previous studies showed that stress reduces the phosphorylation of G3BP1 and increase the formation of SGs [46]. Thus, we investigated whether VRK2 has a role in this reduction of G3BP1 phosphorylation under the stressful environment. We found that the protein level of VRK2 as well as the phosphorylation of G3BP1 was significantly reduced under the treatment with SA (Fig. 3A–C, and Supplemental Material). Also, when we rescued the level of VRK2 by the overexpression of Flag-VRK2 under SA-mediated stress, the phosphorylation of G3BP1 was significantly rescued (Fig. 3D–E, and Supplemental Material). However, when we overexpressed kinase dead form of VRK2 (Flag-VRK2 KD), the phosphorylation of G3BP1 was not rescued (Fig. 3D–E, and Supplemental Material). These results demonstrate that diminished phosphorylation of G3BP1 during the stressful environment is largely due to the reduction of VRK2.

### The level of VRK2 is regulated by RNF144A-mediated proteasomal degradation

**Fig. 4:**
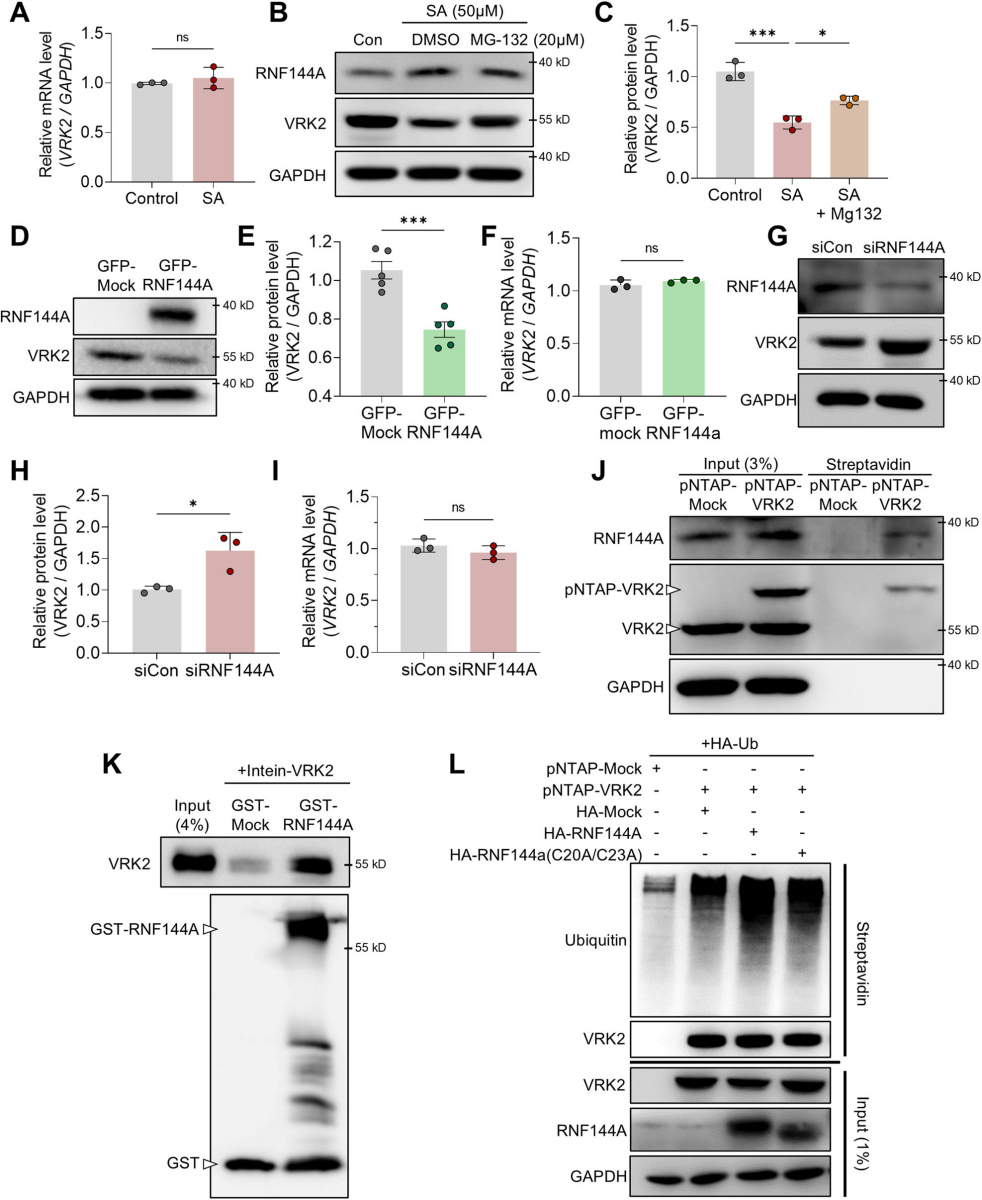
VRK2 is downregulated by RNF144A through proteasomal degradation. **A** mRNA level of *VRK2* was measured in stress-induced U2OS cells. U2OS cells were treated with SA to induce stress (50 μM, 8 h). mRNA level of *GAPDH* was used for normalization (*n* = 3). Representative immunoblot (**B**) and the quantification (**C**) of VRK2 under stress and inhibition of proteasomal degradation. Stress was induced in U2OS cells with SA (50 μM, 8 h) and proteasomal degradation was inhibited using MG-132 (20 μM, 8 h). GAPDH was used for normalization (*n* = 3). Representative immunoblot (**D**) and the quantification of VRK2 protein level (**E**) and *VRK2* mRNA level (**F**) in RNF144A-excessive U2OS cells. U2OS cells were transfected with GFP-RNF144A and were incubated for 24 h. Protein level of GAPDH (*n* = 5) and mRNA level of *GAPDH* (*n* = 3) was used for normalization. Representative immunoblot (**G**) and the quantification of VRK2 protein level (**H**) and *VRK2* mRNA level (**I**) in RNF144A-deficient U2OS cells. U2OS cells were transfected with siRNF144A and were incubated for 24 h. Protein level of GAPDH (*n* = 3) and mRNA level of *GAPDH* (*n* = 3) were used for normalization. **J** Representative immunoblot of pNTAP-streptavidin pull down assay performed with pNTAP-Mock or pNTAP-VRK2 transfected U2OS cells. GAPDH was used for normalization. **K** Representative immunoblot of GST pull down assay performed with recombinant GST-RNF144A and Intein-VRK2. **L** Representative immunoblot of poly-ubiquitination assay. GAPDH was used for normalization. n.s., not significant, *p ≤ 0.05, **p ≤ 0.01, ***p ≤ 0.001, ****p ≤ 0.0001; unpaired Student’s t test was performed for (**A**, **C**, **E**, **F**, **H**, **I**). The “n” represents the number of independent experiments. Error bars indicate SDs.

We next examined how the protein level of VRK2 is downregulated under the stress. When we measured the mRNA level of *VRK2* through real time quantitative polymerase chain reaction (RT-qPCR), the treatment with SA did not induce any changes to the mRNA level (Fig. 4A). This indicates that the level of VRK2 is not regulated at the transcriptional level. Interestingly, when we additionally treated MG-132, a proteasome inhibitor, the level of VRK2 was significantly rescued (Fig. 4B, C, and Supplemental Material). This implies that VRK2 is downregulated through proteasomal degradation under stress.

The process of proteasomal degradation starts with ubiquitin-protein ligase, also known as the E3 ligase [47]. Once the E3 ligase transfers the ubiquitin from the E2 ligase to the substrate protein, an elongated chain of ubiquitin that is recognized by the proteasome is formed [47]. Our group previously discovered that RNF144A mediates proteasomal degradation of VRK3 as an E3 ligase, and suggested its potential function as an E3 ligase of VRK2 [43]. Thus, we assessed whether RNF144A mediates the proteasomal degradation of VRK2. When we overexpressed RNF144A by GFP-RNF144A, the protein level of VRK2 was significantly decreased while its mRNA level was not changed (Fig. 4D–F, and Supplemental Material). Gradual overexpression of RNF144A gradually decreased the level of VRK2 (Supplementary Fig. 3A). On the other hand, when the level of RNF144A was knocked down by RNAi system, the protein level of VRK2 was increased while its mRNA level remained steady (Fig. 4G–I, and Supplemental Material). These results show that the level of VRK2 is surely affected by the level of RNF144A. Surprisingly, the downregulation of VRK2 by the overexpression of RNF144A was rescued by the treatment with MG-132, which further associates RNF144A’s function as an E3 ligase of VRK2 (Supplementary Fig. 3B and Supplemental Material). Thus, we explored whether RNF144A interacts with VRK2. Through pNTAP-streptavidin pull down assay with U2OS cells, we detected an interaction between VRK2 and RNF144A (Fig. 4J and Supplemental Material). We confirmed the interactions between VRK2 and RNF144A through immunoprecipitation of Flag-VRK2 with Flag antibody (Supplementary Fig. 3C). We further demonstrated a direct interaction between VRK2 and RNF144A through GST pull down assay (Fig. 4K and Supplemental Material). To confirm whether RNF144A could transfer ubiquitin to VRK2 as an E3 ligase, we performed poly-ubiquitination assay. Intriguingly, the ubiquitination of VRK2 was significantly increased by the overexpression of RNF144A (Fig. 4L and Supplemental Material). However, this increase in the ubiquitination was no longer seen when the E3 ligase activity of RNF144A was inhibited by the mutation (C20A/C23A) (Fig. 4L and Supplemental Material). Overall, these results demonstrate that RNF144A mediates proteasomal degradation of VRK2 as an E3 ligase.

### RNF144A-VRK2-G3BP1 axis in SA-mediated SG formation

**Fig. 5:**
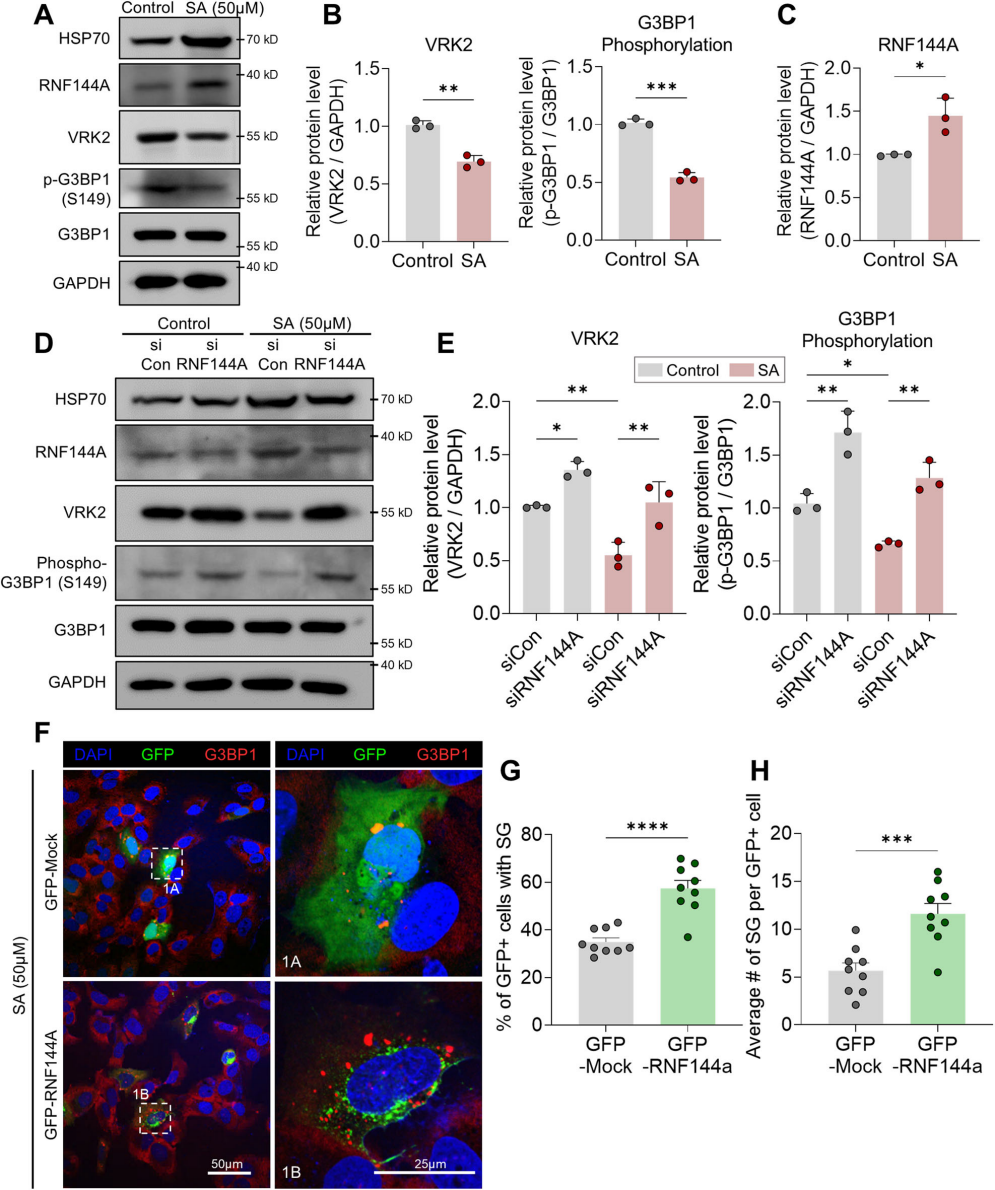
RNF144A-VRK2-G3BP1 axis participates in SA-mediated SG formation. Representative immunoblot (**A**) and the quantification of VRK2, G3BP1 phosphorylation (**B**), and RNF144A (**C**) in stress-induced U2OS cells. The stress was induced with SA (50 μM, 8 h). HSP70 was used as the marker for SA-induced stress. The phosphorylation of G3BP1 is normalized to total G3BP1 and GAPDH was used as loading control (*n* = 3). Representative immunoblot (**D**) and the quantification of VRK2 and G3BP1 phosphorylation (**E**) in stress-induced and RNF144A-deficient U2OS cells. U2OS cells were transfected with siRNF144A and the stress was induced with SA (50 μM, 8 h). HSP70 was used as the marker for SA-induced stress. The phosphorylation of G3BP1 is normalized to total G3BP1, and GAPDH was used as loading control (*n* = 3). **F** Representative image of SG formation in RNF144A-overexpressed U2OS cells. U2OS cells were transfected with GFP-RNF144A, and the stress was induced by the treatment with SA (50 μM, 2 h). SGs were stained with G3BP1 (red), and the nuclei of cells were stained with DAPI (blue). Scale bar = 50 µm. The formation of SG in single cell is shown through up-scaled image (1 A and 1B, Scale bar = 25 µm). Proportion of U2OS cells with SGs (**F**) and average number of SGs per U2OS cells (**G**) were quantified in control or RNF144A-excessive U2OS cells (*n* = 9). * *p* ≤ 0.05, ** *p* ≤ 0.01, *** *p* ≤ 0.001, **** *p* ≤ 0.0001; unpaired Student’s t test was performed for (**B**, **C**, **G**, **H**); two-way ANOVA with Tukey’s multiple comparison test was performed for (**E**). The “n” represents the number of independent experiments. Error bars indicate SDs.

Since RNF144A regulates the protein level of VRK2, we checked whether such regulation exists under the stressful conditions. Similar to previous results, the amount of VRK2 as well as the phosphorylation of G3BP1 was diminished under the stress induced by SA (Fig. 5A, B, and Supplemental Material). Contradictorily, the level of RNF144A was substantially increased by the stress (Fig. 5C), which was confirmed through time dependent experiments (Supplementary Fig. 4A–D, and Supplemental Material). Interestingly, the level of RNF144A, VRK2, and the phosphorylation of G3BP1 was also changed in other cancer cells treated with SA, such as A549 lung cancer cells and MDA-MB-231 breast cancer cells (Supplementary Fig. 4E, F, and Supplemental Material). To confirm if the increase in RNF144A directly mediates the loss of VRK2 during stress, we knocked down RNF144A under the stress. Interestingly, the level of VRK2, as well as the phosphorylation of G3BP1, was rescued when RNF144A was depleted with RNAi system (Fig. 5D, E, and Supplemental Material). Furthermore, we checked whether the regulation of VRK2 by RNF144A during the stress affects the formation of SGs. When RNF144A was overexpressed by the transfection with GFP-RNF144A, both the proportion of cells with SGs and the average number of SGs per cell were significantly increased (Fig. 5F–H). Thus, an increase in RNF144A elevated the formation of SGs. Collectively, these data reveal an existence of RNF144A-VRK2-G3BP1 axis that participates in the formation of SGs.

### RNF144A-VRK2-G3BP1 axis in cisplatin-mediated SG formation

**Fig. 6:**
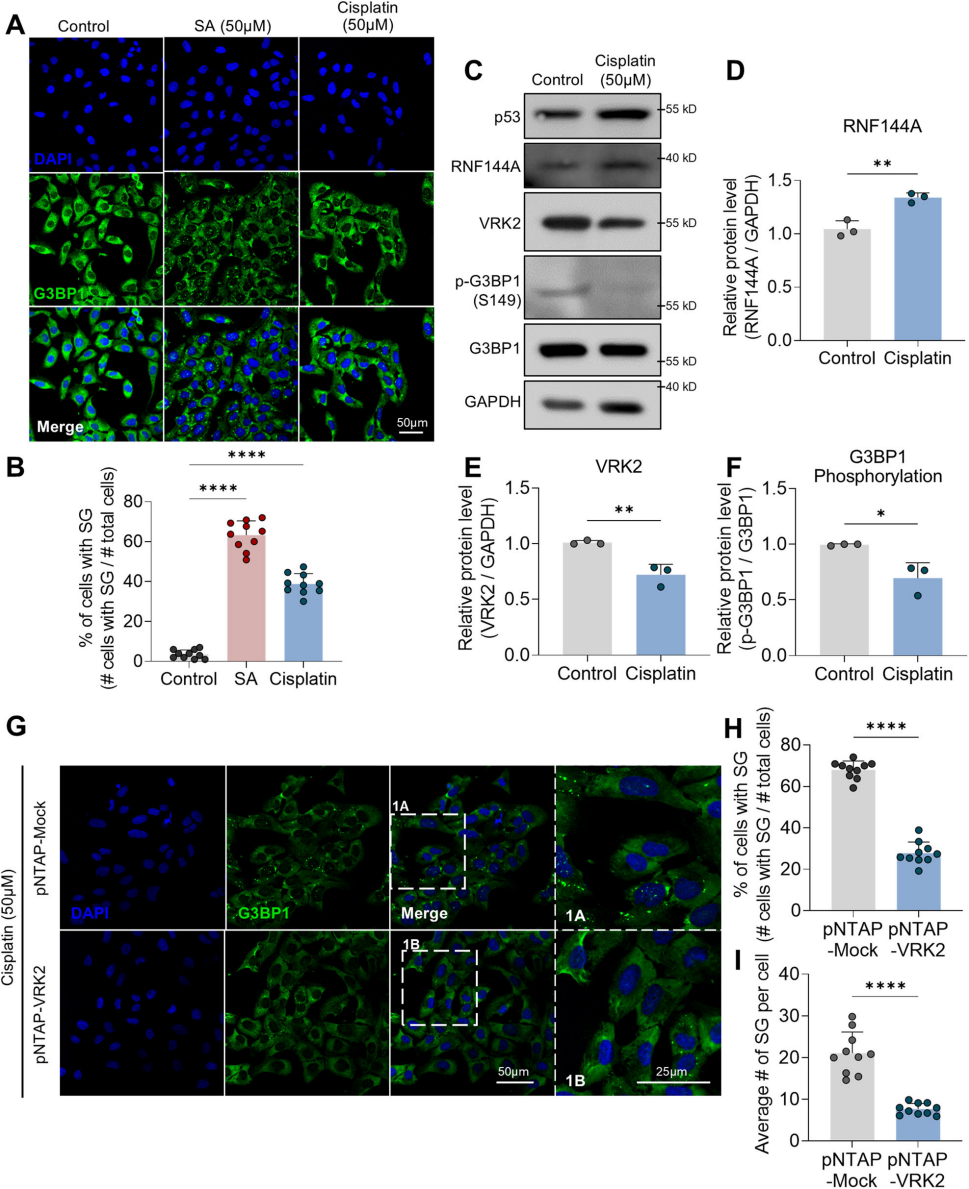
RNF144A-VRK2-G3BP1 axis participates in cisplatin-mediated SG formation. **A** Representative image of SG formation in U2OS cells. The stress was induced by the treatment with cisplatin (50 μM, 2 h). SGs were stained with G3BP1 (green), and the nuclei of cells were stained with DAPI (blue). Scale bar = 50 µm. **B** Proportion of U2OS cells with SGs were quantified in U2OS cells treated with cisplatin (*n* = 10). Representative immunoblot (**C**) and the quantification of RNF144A (**D**), VRK2 (**E**), and G3BP1 phosphorylation (**F**) in stress-induced U2OS cells. The stress was induced with cisplatin (50 μM, 8 h). p53 was used as the marker for cisplatin-induced stress. The phosphorylation of G3BP1 is normalized to total G3BP1, and GAPDH was used as loading control (*n* = 3). **G** Representative image of SG formation in VRK2-excessive U2OS cells. U2OS cells were transfected with pNTAP-VRK2, and the stress was induced by the treatment with cisplatin (50 μM, 2 h). SGs were stained with G3BP1 (green), and the nuclei of cells were stained with DAPI (blue). Scale bar = 50 µm. The formation of SG in single cell is shown through up-scaled image (1 A and 1B, Scale bar = 25 µm). Proportion of U2OS cells with SGs (**H**) and average number of SGs per U2OS cells (**I**) were quantified in control or VRK2-excessive U2OS cells (*n* = 10). *p ≤ 0.05, **p ≤ 0.01, ***p ≤ 0.001, ****p ≤ 0.0001; ordinary one-way ANOVA with Tukey’s multiple comparison test was performed for (**B**); unpaired Student’s t test was performed for (**D**, **E**, **F**, **H**, **I**). The “n” represents the number of independent experiments. Error bars indicate SDs.

Although different strategies for cancer treatment are being developed, chemotherapy is still one of the most widely used anti-cancer therapy [48]. One of the problems with the anti-cancer chemotherapy is that some chemotherapeutic agents induce the formation of SGs in cancer cells [18, 19]. The SGs promote proliferation and survival of cancer cells through various mechanisms [49]. Thus, the sensitivity to the chemotherapy may fall over time [18, 19]. Unfortunately, the mechanism behind the formation of SGs induced by the chemotherapy is still to be found. Hence, we examined if the RNF144A-VRK2-G3BP1 axis participates in chemotherapy-mediated SG formation. In accordance to previous reports, we were able to observe the SGs when we treated the cisplatin [24], a widely used chemotherapeutic agents for osteosarcoma, to U2OS cells (Fig. 6A). Although the proportion of cells with SGs were lower than that of U2OS cells treated with SA, there was still significant amount of cells with SGs (Fig. 6B). We, then, checked if the RNF144A-VRK2-G3BP1 axis is regulated by the treatment with cisplatin. Similar to the previous results with SA, the treatment with cisplatin increased the level of RNF144A and decreased the level of VRK2, as well as the phosphorylation of G3BP1 (Fig. 6C–F, Supplementary Fig. 4G, and Supplemental Material). Also, when we rescued the level of VRK2 in U2OS cells treated with cisplatin, both the proportion of cells with SGs and the average number of SGs per cell were significantly decreased (Fig. 6G–I). These data show that RNF144A-VRK2-G3BP1 axis participates in the formation of cisplatin-mediated SGs.

**Fig. 7:**
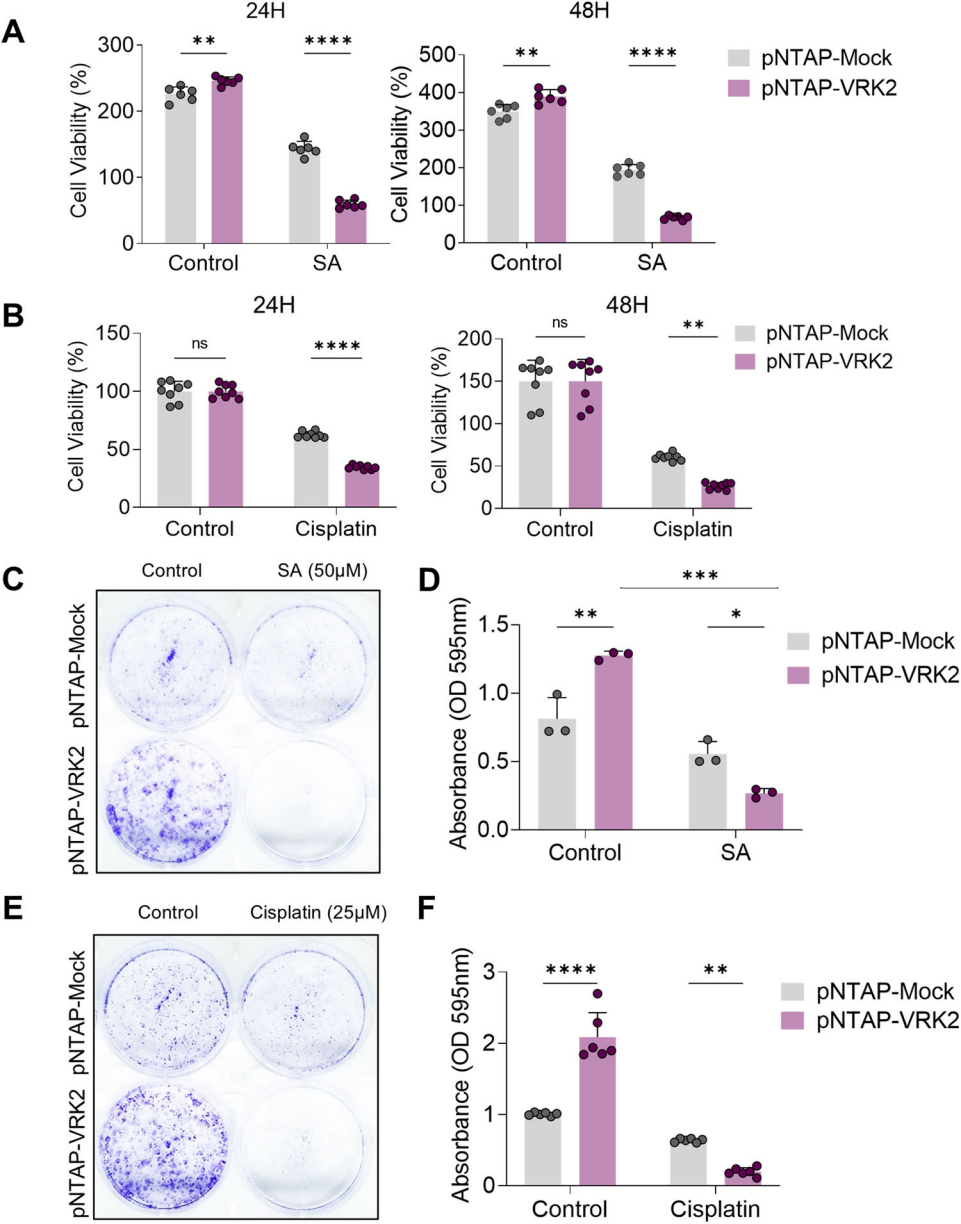
RNF144A-VRK2-G3BP1 axis sensitizes osteosarcoma cell to stress. **A**, **B** The cell viability of pNTAP-VRK2 stably expressing U2OS cells was measured through CCK-8 assay. The stress was induced with 50 μM of SA (**A**) or 50 μM of cisplatin (**B**) for 24 h or 48 h (*n* = 6, *n* = 8, respectively). Representative image (**C**) and the quantification (**D**) of colony formation assay performed with pNTAP-VRK2 stably expressing U2OS cells. pNTAP-VRK2 stably expressing U2OS cells were treated with SA (50 μM) from 4th day to 7th day (*n* = 3). Representative image (**E**) and the quantification (**F**) of colony formation assay performed with pNTAP-VRK2 stable U2OS cells. pNTAP-VRK2 stably expressing U2OS cells were treated with cisplatin (25 μM) from 4th day to 7th day (*n* = 3). n.s., not significant, *p ≤ 0.05, **p ≤ 0.01, ***p ≤ 0.001, ****p ≤ 0.0001; two-way ANOVA with Tukey’s multiple comparison test was performed for (**A**, **B**, **D**, **F**). The “n” represents the number of independent experiments. Error bars indicate SDs.

We next assessed if modulating the RNF144A-VRK2-G3BP1 axis could maximize the cancer cell’s sensitivity to stress or chemotherapy. To modulate RNF144A-VRK2-G3BP1 axis in U2OS cells, we generated cell lines that stably express either pNTAP-Mock or pNTAP-VRK2. Then, we used SA or cisplatin to induce the stress in these stable cell lines. When we measured cell survival through CCK-8 assay, pNTAP-VRK2 U2OS cells responded more sensitively to SA when compared to pNTAP-Mock U2OS cells (Fig. 7A). The cell viability of pNTAP-VRK2 U2OS cells were worse than that of pNTAP-Mock U2OS cells at both 24 and 48 h of SA treatment (Fig. 7A). Similar to SA, the treatment with cisplatin induced greater decrease in the cell viability of pNTAP-VRK2 U2OS cells than that of pNTAP-Mock U2OS cells (Fig. 7B). The knockdown of VRK2 also altered the cell viability of A549 lung cancer cells and MDA-MB-231 breast cancer cells (Supplementary Fig. 4H and I). The knockdown of VRK2 desensitized both types of cancer cells to the stress, increasing their viability even under the SA-mediated stress (Supplementary Fig. 4H and I). The reduction in the resistance to the stress was also confirmed with the colony formation assay. Since VRK2 participates in cell cycle [41], stable overexpression of VRK2 increased the rate of colony formation under normal condition (Fig. 7C, E). However, when either SA or cisplatin was treated, pNTAP-VRK2 cells showed significantly less resistance to the stress (Fig. 7D, F). Overall, these data implicate that the inhibition of SG formation by altering the RNF144A-VRK2-G3BP1 axis can sensitize various cancer cells to stressful environment, leading to their death.

## Discussion

Growing evidence shows the significance of SGs in the progression of cancer. SGs promote cancer cell proliferation and survival in response to the anti-cancer drug allowing the cancer cells to better survive from the drug treatment [18]. Although its importance, the mechanism of the SG formation in cancer cell remains to be fully understood. Here, we revealed a novel SG assembly pathway consisting of RNF144A, VRK2, and G3BP1 in cancer cell under stress.

In U2OS osteosarcoma cells, we discovered that VRK2 phosphorylates Ser149 of G3BP1 and negatively regulates SG assembly. The treatment with SA reduced the level of VRK2 and the phosphorylation at Ser149 of G3BP1, further promoting the formation of SGs. The level of VRK2 was downregulated by RNF144A-mediated proteasomal degradation under stress. Interestingly, the level of RNF144A was significantly increased in response to the stress. These results demonstrate the presence of RNF144A-VRK2-G3BP1 axis that regulates stress-mediated SG formation in U2OS cells. Still, how the level of RNF144A increases under the stress remains unsolved. Previous reports showed that the expression of RNF144A, one of p53 target genes, is increased in response to DNA damage [50]. Under the DNA damage, mouse double minute 2 homolog (MDM2) that mediates the degradation of p53 is displaced from p53, increasing the protein level of p53 [51]. In return, p53 further increases the expression of RNF144A, although a specific mechanism was not revealed yet [50]. Similar to previous reports, we have found that the protein level of p53 was significantly increased in U2OS cells treated with SA (Supplementary Fig. 5 and Supplemental Material). Thus, we could speculate that the increase in the level of RNF144A under the stress is mediated by an increase in the level of p53.

We, then, checked if RNF144A-VRK2-G3BP1 axis participates in the chemotherapy-mediated SG formation. We observed that the treatment with cisplatin induced the formation of SGs in U2OS cells. We also confirmed an increase in the level of RNF144A and a decrease in the level of VRK2 as well as the phosphorylation of its substrate, G3BP1. Additionally, cisplatin-mediated SG formation was significantly reduced by the overexpression of VRK2. These results illustrate that RNF144A-VRK2-G3BP1 axis also regulates cisplatin-mediated SG formation in U2OS cells. Apart from cisplatin, osteosarcoma is often treated with etoposide, another chemotherapeutic agent that induces DNA damage [52, 53]. Previous report showed that the treatment with etoposide induces the formation of SGs in glioma cells [24]. Similar to our scheme, these glioma cells were sensitized to etoposide when SG formation was inhibited [24]. Unfortunately, the formation of SG was barely induced in U2OS cells by the treatment with etoposide (Supplementary Fig. 6A). Also, inhibiting the formation of SG by increasing the level of VRK2 did not affect the U2OS cells’ resistance to etoposide (Supplementary Fig. 6B and C). This indicates that RNF144A-VRK2-G3BP1 axis may regulate the formation of SGs during specific type of stress, which is an interesting topic to study in the future.

We also discovered that increasing the level of VRK2, and thus the inhibition of the SG formation, reduced U2OS cells’ resistance to both SA and cisplatin. Both the viability and the colony formation rate of U2OS cells were significantly decreased by the overexpression of VRK2 during the treatment with SA or cisplatin. These data indicate that inhibiting the SG formation through RNF144A-VRK2-G3BP1 axis could possibly provide effective treatment strategy to overcome the resistance of cancer to anti-cancer chemotherapy. These data also implicate that the distinction between patients’ sensitivity to the chemotherapy may come from the heterogeneous expression of RNF144A-VRK2-G3BP1 axis among the cancer patients. Recently, heterogeneity in tumor has been suggested as the cause of the distinction in resistance to anti-cancer therapies [54]. Several studies have compared the molecular signatures among the cells or the patients that show different sensitivity to chemotherapy [55, 56]. These publicly available gene expression data set (GEO accession: GSE98230 and GSE154540) also showed that the expression of RNF144A and VRK2 has correlation to both cells’ and patients’ sensitivity to chemotherapy (Supplementary Fig. 7). Thus, the patient’s resistance to anti-cancer chemotherapy, like cisplatin, could be due to increased expression of RNF144A that promotes the formation of SGs through VRK2 degradation. Overall, our study demonstrated an existence of RNF144A-VRK2-G3BP1 axis that regulates the formation of SGs in cancer cell under stress, and that modulating the level of axis could possibly maximize the effect of anti-cancer chemotherapy.

## Materials and methods

### Plasmid construction

The coding region of human *G3BP1* (Accession #: NM_005754.3) was amplified by PCR from the cDNAs of U2OS cells using the primers that was synthesized from Macrogen (Marcogen, Seoul, Republic of Korea) (Forward primer: 5′-AAG AAT TCA ATG GTG ATG GAG AAG C-3′; Reverse primer: 5′-AAG GAT CCC TAC TGC CGT GGC GC-3′). The PCR products were cloned into pGEX-4T3 (Amersham Biosciences, Piscataway, NJ, RRID:Addgene_1406). GST-G3BP1 (S149A) mutant form was amplified from GST-G3BP1 plasmid. Two fragments of G3BP1 generated with two different set of primers were amplified as one fragment using Forward primer-1 and Reverse primer-2 (Forward primer-1: 5′-AAG AAT TCA ATG GTG ATG GAG AAG C-3′; Reverse primer-2: 5′-TAC TTC TTC TTC AGC CTC CTC CTG AGG-3′; Forward primer-2: 5′-CCT CAG GAG GAG GCT GAA GAA GAA GTA-3′; Reverse primer-2: 5′-AAG GAT CCC TAC TGC CGT GGC GC-3′). The full-length coding sequence of G3BP1 mutant form was cloned into pGEX-4T3 vector. VRK2 plasmids used in this study are described in previous reports [39, 57]. Briefly, the coding region of human *VRK2* (Accession #: NM_001130480.2) was amplified by PCR from the cDNAs of U2OS cells (Forward primer: 5′-TAT GGA CTA CAA GGA CGA CGA TGA CAA AG-3′; Reverse primer: 5′-CTA GCT TTG TCA TCG TCG TC C TTG TAG TCC A-3′). The PCR products were cloned into pNTAP-B (Agilent Technologies, California, USA), pPROEX-Hta (Invitrogen, California, USA), pEGFP-C1 (BD Biosciences, California, USA), pTYB2 (Agilent Technologies, California, USA), and pFLAG-CMV2 (RRID:Addgene_15697) plasmids. VRK2 kinase dead form (VRK2 KD) was amplified from pFLAG-VRK2 and was cloned into pFLAG-CMV2 plasmid (Forward primer: 5′-AAG CTA GCA TGG CAC CAA GAA GAA AAG AGA AA-3’; Reverse primer: 5′-AAC TCG AGA TCT GCC TTT GTC TCT TCA CTC A-3′). RNF144A plasmids used in this study (pEGFP-C1 RNF144A, pcDNA3.1-HA RNF144A, and pcDNA3.1-HA RNF144A(C20A/C23A)) were kindly gifted by Seung Hyun Han (Hesed Bio, Pohang, Republic of Korea)[43].

### Cell culture, drug treatment, and generation of stable cell line

U2OS (Korean Cell Line Bank, RRID:CVCL_0042), HEK293A (Korean Cell Line Bank, RRID:CVCL_6910), A-549 (Korean Cell Line Bank, RRID:CVCL_0023), and MDA-MB-231 (Korean Cell Line Bank, RRID:CVCL_0062) cells were cultured with Dulbecco’s Modified Eagle’s Medium (DMEM, HyClone, UT, USA) that is supplemented with 10% fetal bovine serum (FBS, HyClone, UT, USA) and 1% penicillin G and streptomycin (Welgene, Daegu, Republic of Korea). The cells were cultured in a humidified atmosphere that contained 5% CO_2_ at 37 °C. None of our cell lines were listed as commonly misidentified cell lines by the International Cell Line Authentication Committee (ICLAC). Prior to the experiments, they were also authenticated through PCR-based short tandem repeat assays. Mycoplasma contaminations were confirmed using MycoAlert® Mycoplasma Detection Kits (Lonza, Basel, Switzerland). U2OS cells were treated with 50 μM of sodium arsenite (SA, Sigma–Aldrich, California, USA) from 2 h to 12 h to induce stress depending on the experiments. U2OS cells were also treated with 50 μM of cisplatin (Sigma–Aldrich, California, USA) and 25 μM of etoposide (Sigma–Aldrich, California, USA) from 2 h to 12 h depending on the experiments.

To generate U2OS cell line that stably expresses VRK2, U2OS cells were transfected with either pNTAP-Mock or pNTAP-VRK2 plasmids using Neon Transfection System (Invitrogen, California, USA). After 24 h of incubation, transfected cells were selected through the treatment with G418 disulfate salt (800 μg/ml, Sigma–Aldrich, California, USA). G418 disulfate salt containing media were changed every 2 days. The overexpression of VRK2 was checked by Western blot.

### pNTAP-streptavidin pull down assay

U2OS cells were transfected with either pNTAP-Mock or pNTAP-VRK2 through Neon Transfection System. After 24 h of incubation, the cells were lysed in RIPA buffer (10 mM Tris-HCl, 1 mM EDTA, 0.1% SDS, 2.4 mM sodium deoxycholate, and 1% Triton X-100 (Sigma-Aldrich, California, USA)) with protease inhibitor cocktail (ThermoFisher, IL, USA) followed by sonication. 1 mg of cell lysates was incubated with streptavidin agarose resin (ThermoFisher, IL, USA) on a rotator for 12 h at 4 °C. After the incubation, the resins were washed three times with RIPA buffer and 2X sample buffer (0.6% 1 M Tris, 50% Glycerol, 10% SDS, 0.5% 2-Mercaptoethanol, and 1% Bromophenol blue) was added to the resins. The resins were then heated at 98 °C for 9 min, being subjected to vortex in between. After centrifugation at 15000 rpm 4 °C, the supernatants were subjected to sodium dodecyl sulfate-polyacrylamide gel electrophoresis (SDS-PAGE) and immunoblotting.

### Immunoprecipitation

HEK293A cells were transfected with FLAG-Mock, FLAG-VRK2, GFP-Mock, and GFP-RNF144A. After 24 h of incubation, transfected cells were suspended in lysis buffer (50 mM Tris (pH7.5), 30 mM NaCl, 2 mM EDTA, 1% Triton X-100 and protease inhibitor) and were disrupted using sonication. 1 mg of cell lysates was incubated with protein A-agarose beads (Roche, Basel, Switzerland) and Anti-Flag antibody (Sigma–Aldrich, California, USA) overnight at 4 °C on a rotator. After 12 h of incubation, beads were washed three times with lysis buffer. 1 mM DTT and 2x sample buffer were added to the beads, and samples were heated at 98 °C for 8 min. The samples were analyzed through SDS-PAGE and immunoblotting.

### GST pull down assay

GST-pull down assay was performed following the methods from the previous study[43]. Briefly, GST or GST-tagged recombinant proteins were mixed with either His-tagged recombinant proteins or Intein-tagged recombinant proteins in cell lysis buffer (20 mM Tris, 150 mM NaCl, 1 mM EDTA, and 0.5% Triton-X). The mixture was then added to Glutathione Sepharose 4B beads (Cytiva, MA, USA) and was further incubated for overnight at 4 °C on a rotator. After the incubation, Glutathione Sepharose 4B beads were washed three times with cold lysis buffer. After washing, 2x sample buffer and 1 mM DTT were added, and the sample was heated at 95 °C for 7 min. The mixture was then subjected to SDS-PAGE and immunoblotting.

### In vitro kinase assay

For in vitro kinase assay, 1 μg of recombinant GST-tagged proteins (or His-VRK2 alone) and 1 μg His-VRK2 recombinant protein were incubated at 37 °C in a 20 μl of reaction mixture containing kinase buffer (20 mM Tris-HCl, 5 mM MgCl2, 0.5 mM dithiothreitol, 150 mM KCl, and [γ-^32^P] ATP). After 30 min of incubation, the reactions are heated at 95 °C for 5 min, and then subjected to SDS-PAGE, and were further visualized with autoradiography. Gel from SDS-PAGE were stained with Coomassie brilliant blue solution to quantify the amount of protein loaded.

### RNA interference

U2OS cells were transfected with small interfering RNAs (siRNAs) using Neon Transfection System or by Lipofectamine 2000 (Invitrogen, California, USA) according to the manufacturer’s instruction. The siRNAs that were used in this study are as follows: si-control (Bioneer; 5′-CCU ACG CCA CCA AUU UCG U-3′), si-VRK2 (Bioneer; 5′-CAC AAU AGG UUA AUC GAA A-3′), and si-RNF144A (Horizon Discovery; 5′-GUG CAA AGC CUG CCG UAU G-3′)

### SDS-PAGE and immunoblotting

SDS-PAGE was performed as previously described [58]. Briefly, U2OS cells from different experiments were lysed in RIPA buffer and disrupted through sonication. A total of 30 μg of cell lysates was mixed with 5x sample buffer (0.6% 1 M Tris, 50% Glycerol, 10% SDS, 0.5% 2-Mercaptoethanol, and 1% Bromophenol blue) to generate loading samples. The samples were loaded onto the Western blot gel and resolved in electrophoresis chambers (Bio-Rad, California, USA). Then, the proteins in the gel were transferred to nitrocellulose membranes (Pall Corporation, NY, USA). For immunoblotting, the membranes were incubated with different primary antibodies for 12 h at 4 °C followed by secondary antibody for 2 h at room temperature. The membranes were visualized with the LAS-4000 system (FUJIFILM, Tokyo, Japan) after treating the membrane with enhanced chemiluminescent (ECL) solution.

### Antibody

For primary antibodies in Western blot analysis, Anti-G3BP1 (1:1000; Santa Cruz Biotechnology Cat# sc-365338, RRID:AB_10846950), Anti-VRK2 (1:400; (Santa Cruz Biotechnology Cat# sc-365199, RRID:AB_10707689), Anti-GAPDH (1:1000; Santa Cruz Biotechnology Cat# sc-47724, RRID:AB_627678), Anti-GST (1:1000; Cell Signaling Technology, #2625), Anti-phospho-G3BP1 (Ser149) (1:500, Invitrogen, PA5-64827), Anti-Flag (1:1000; Cell Signaling Technology, #2368), Anti-HSP70 (1:300; Santa Cruz, sc-24), Anti-RNF144A (1:200; LSBio, LS-C162648), Anti-Ubiquitin (1:1000; Santa Cruz, sc-8017), and Anti-p53 (1:2000; Santa Cruz, sc-126) were used. For secondary antibodies in Western blot analysis, horseradish peroxidase (HRP)-conjugated Anti-Mouse IgG (1:5000; Invitrogen, 31430) and HRP-conjugated Anti-Rabbit IgG (1:5000; Promega, W4018) were used. For primary antibodies in immunocytochemistry, Anti-G3BP1 (1:100; Santa Cruz, sc-365338) was used to stain SGs. For secondary antibodies in immunocytochemistry, Anti-Mouse IgG conjugated with Alexa Fluor™ 488 (1:1000; Thermo Fisher Scientific Cat# A-11001, RRID:AB_2534069) or Alexa Fluor™ 594 (1:1000; Invitrogen, A-11005) was used.

### Stress granule analysis

To quantify SGs, SA-treated U2OS cells were fixed in 4% paraformaldehyde (PFA)-PBS solution for 20 min at room temperature. After three washes with phosphate buffered saline (PBS), the cells were permeabilized with 0.5% Triton X-100 dissolved in PBS for 10 min at room temperature, blocked with 5% FBS-PBS solution for 2 h, and incubated with primary antibody (Anti-G3BP1) for 12 h at 4 °C. Then, the cells were labeled with secondary antibodies conjugated with Alexa 594 or Alexa 488 in PBS for 1 h and were mounted with Dako Fluorescent Mounting Medium (Agilent, Santa Clara, USA). Images were obtained using a FV3000 Confocal Laser Scanning microscope (Olympus, Tokyo, Japan) and Coherent® High Performance OBIS™ laser with wavelengths of 405 nm, 488 nm, and 561 nm. The FV31S-SW fluoviewer software was used to export the images. Proportion of cells with SGs and the average number of SGs per cell were measured from arbitrarily selected fields of view that contained more than 30 cells. The number of cells and the SGs were counted and analyzed using ImageJ software (NIH, Bethesda, USA, RRID:SCR_003070).

### RNA extraction and RT-qPCR

The RNA from U2OS cells was extracted using TRI-solution (Bio Science Technology, VA, USA) according to the manufacturer’s instructions. Briefly, the cells were homogenized in the TRI-solution before adding one-fifth volume of chloroform. The samples were incubated at room temperature for 10 min before the centrifugation at 15,000 rpm, at 4 °C, for 10 min. Then, the supernatant was mixed with an equal volume of isopropanol and incubated in ice for 10 min. The samples were then centrifuged at 15,000 rpm, at 4 °C, for 10 min. The RNA pellets were washed in ethanol and dissolved in DEPC-treated water. Isolated RNAs from the cell were reverse-transcribed with Improm-II^TM^ Reverse Transcription System (Promega, Wisconsin, USA) following the provider’s instructions. The cDNA from RT-PCR was used to measure the RNA level of cells. FastStart Universal SYBR Green Master (Roche, Basel, Switzerland) was used for the reaction, while StepOnePlus^TM^ Real-Time PCR system (ThermoFisher, IL, USA) was used to measure the mRNA level. Primers for human *VRK2* (Forward primer: 5′-TTT AGC ATA TGA TGA AAA GCC AAA CTA TCA-3′; Reverse primer: 5′-TGA GAC TCT TGA TAT TTC TGT CTT CTC CTT-3’) and human *GAPDH* (Forward primer: 5′-CCA AGG AGT AAG ACC CCT GG-3′; Reverse primer: 5′-AGG GGA GAT TCA GTG TGG TG-3′) is as indicated.

### Poly-ubiquitination assay

HEK293A cells were transfected with pNTAP-Mock, pNTAP-VRK2, HA-Mock, HA-RNF144A, HA-RNF144A C20A/C23A (RING1-dead mutant), and HA-Ubiquitin. After 48 h of incubation, transfected cells were resuspended in RIPA buffer and disrupted using sonication. 2 mg of cell lysates was incubated with streptavidin agarose resin for overnight at 4 °C on a rotator. After 12 h of incubation, resins were washed three times with RIPA buffer. 2x sample buffer was then added to the resins, and samples were heated at 98 °C for 8 min. The samples were subjected to SDS-PAGE and immunoblotting.

### CCK-8 assay and colony formation assay

Either pNTAP-Mock U2OS cells or pNTAP-VRK2 U2OS cells were plated in a 96-well plate with the density of 5 × 10^3^ cells per well and were cultured for 24 h. Then, either SA or cisplatin was treated for indicated times (24 h or 48 h). After the indicated time, 10 μL of CCK-8 solution (Dojindo Molecular Technologies, MD, USA) was added to the media and the cells were incubated at 37 °C for 2 h. The absorbance at 450 nm was measured using a microplate reader (Tecan, Männedorf, Switzerland).

For colony formation assay, either pNTAP-Mock U2OS cells or pNTAP-VRK2 U2OS cells were seeded at a density of 2 × 10^3^ cells in each well of 6-well plate. Six days after the seeding, cells were stained with 0.5% crystal violet in 25% methanol for 1 h. Plates were then washed with PBS to remove excessive dye, and their images were taken using a digital camera (Canon, Japan). Quantitative changes in clonogenicity were determined by extracting colonies with 20% acetic acid and measuring the absorbance at 595 nm.

### Experimental design and statistical analysis

All cell-based data are the results of at least three independent experiments performed with cells from different passage. All microscopy experiments with stress-induced U2OS cells were repeated at least 9 times. The comparison between two groups were statistically analyzed by unpaired Student’s t tests. Comparisons between three or more groups with one independent variable were analyzed by ordinary one-way analysis of variance (ANOVA) with Tukey’s multiple comparison test. When there were two independent variables in the experiment, ordinary two-way ANOVA with Tukey’s multiple comparison test was used for analysis. All quantitative data are presented as means ± SD. *p* values greater than 0.05 was considered not significant. The significance of the statistical analysis is indicated as such: n.s., not significant, *p ≤ 0.05, **p ≤ 0.01, ***p ≤ 0.001, and ****p ≤ 0.0001.

## Supplementary information

Supplementary figures

Original Data

## Data Availability

The data generated in this study are available upon request from the corresponding author.
