# Supplementary material for: RNF144A-VRK2-G3BP1 axis regulates stress granule assembly
Source: Cell Death Discov. 2025 Apr 9;11:158. doi: 10.1038/s41420-025-02460-6 (PMC11982375; doi:10.1038/s41420-025-02460-6)
Supplement: Supplementary file 1 — Supplementary figures [file 41420_2025_2460_MOESM1_ESM.docx]

Supplementary Material for

**RNF144A-VRK2-G3BP1 axis regulates stress granule assembly**

Sung Wook Kim^±^, Jae Lee^±^, Kyung Won Jo, Young-Hun Jeong, Won Sik Shin, and Kyong-Tai Kim*

^±^These authors contributed equally to this work.

*Corresponding authors E-mail: [ktk@postech.ac.kr](mailto:ktk@postech.ac.kr)

**This file includes:**

Figs. S1 to S7


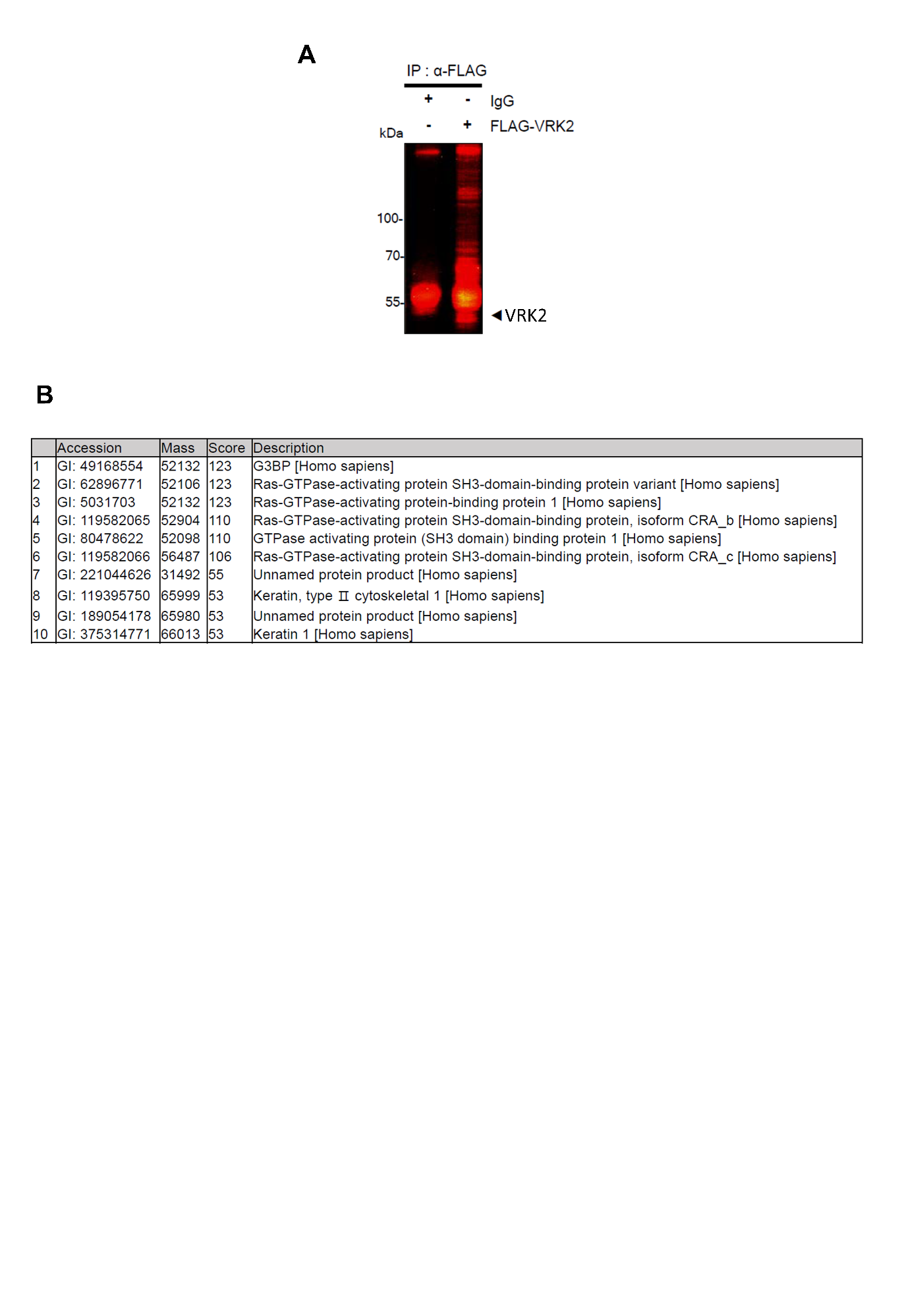


**Fig. S1. G3BP1 is a binding protein that interacts with VRK2.** (**A**) SDS-PAGE (sodium dodecyl sulfate–polyacrylamide gel electrophoresis) gel with the protein band that binds to recombinant Flag-VRK2 identified by immunoprecipitation (IP) analysis was cut out. Black arrow indicates the location of Flag-VRK2. The gel was used to perform mass spectrometry (MS) analysis. (**B**) Results of MS analysis of protein bands (n=1).


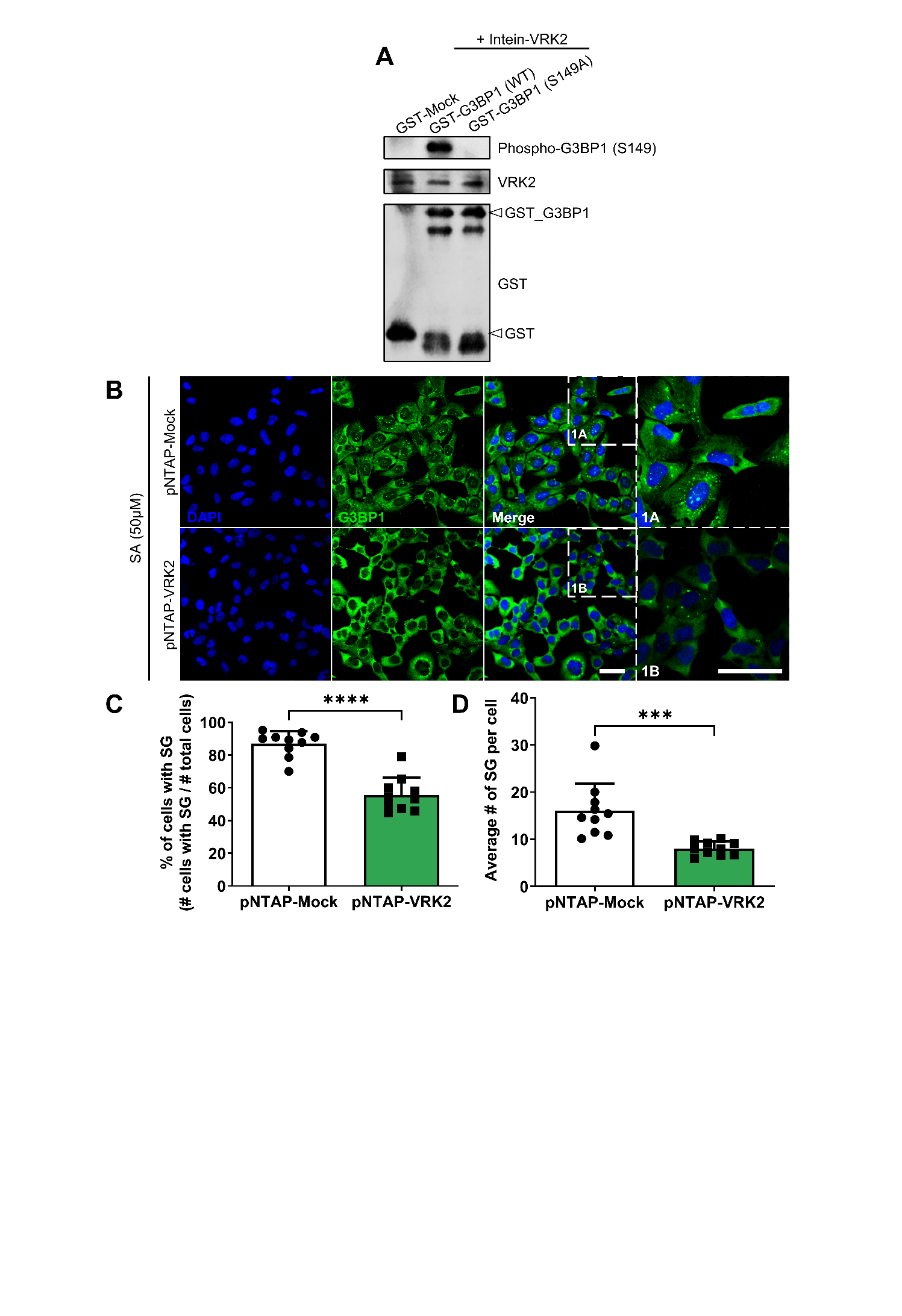


**Fig. S2. VRK2 directly interacts with G3BP1 and regulates SG formation.** (**A**) Representative immunoblot from *in vitro* kinase assay using recombinant GST-G3BP1 and Intein-VRK2; WT: wild type, S149A: mutation of Ser149 to alanine. Cold ATP was used instead of γ-^32^P ATP, and the phosphorylation was detected using antibody. (**B**) Representative image of SG formation in pNTAP-VRK2 stably expressing U2OS cells. The stress was induced by the treatment with SA (50μM, 2 hours). SGs were stained with G3BP1 (green) and the nuclei of cells were stained with Hoechst 33342 (blue). Scale bar = 50µm. The formation of SG in single cell is shown through up-scaled image (1A and 1B, Scale bar = 50µm). (**C,D**) Proportion of U2OS cells with SGs (C) and average number of SGs per U2OS cells (D) were quantified in pNTAP-VRK2 stably expressing U2OS cells (n=10). *** *p* ≤ 0.001, **** *p* ≤ 0.0001; unpaired Student’s t test was performed for (**C,D**). The “n” represents the number of independent experiments. Error bars indicate SDs.


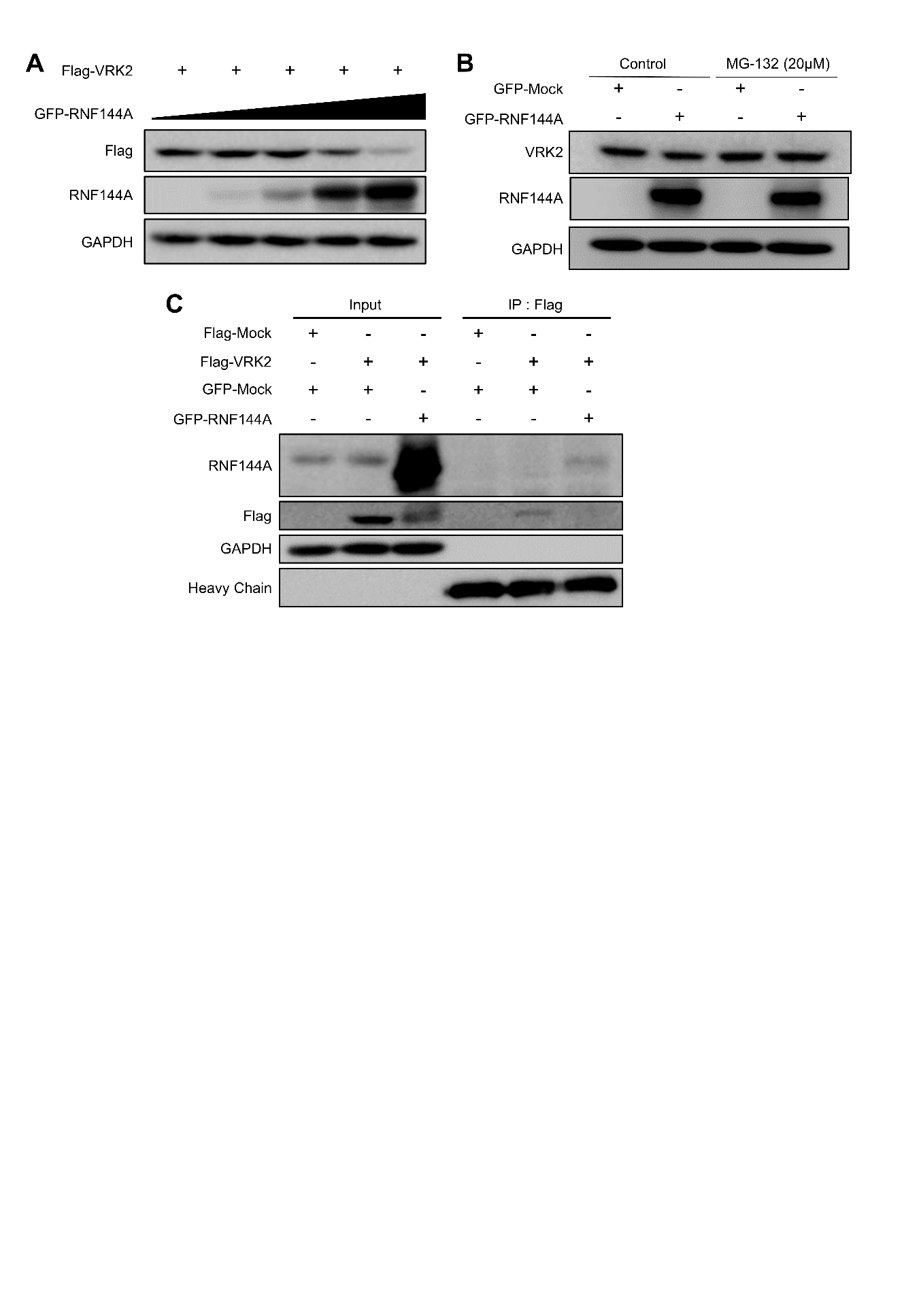


**Fig. S3. RNF144A interacts with VRK2.** (**A**) Representative immunoblot of RNF144A-dependent degradation of VRK2. U2OS cells were transfected with same amount of Flag-VRK2 and increasing amount of GFP-RNF144A. GAPDH was used for normalization. (**B**) Representative immunoblot of VRK2 after the overexpression of RNF144A and inhibition of proteasomal degradation. U2OS cells were transfected with GFP-RNF144A and proteasomal degradation was inhibited using MG-132 (20μM, 8 hours). GAPDH was used for normalization. (**C**) Representative immunoblot from immunoprecipitation of Flag-VRK2. Flag-VRK2 was pull down using Anti-Flag.


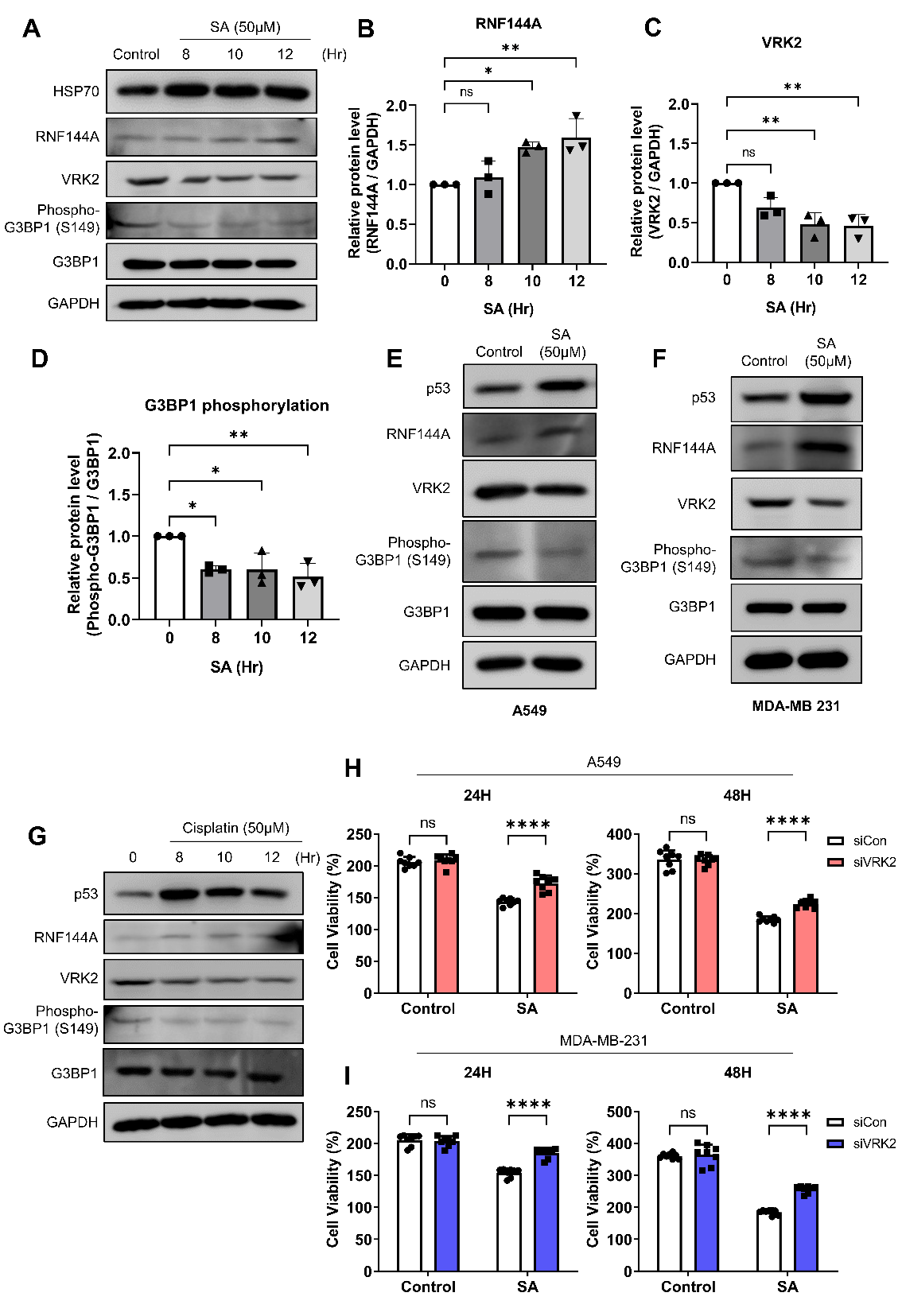


**Fig. S4. Time-dependent changes to RNF144A-VRK2-G3BP1 axis under SA- or cisplatin-mediated stress.** (**A-D**) Representative immunoblot (A) and the quantification of RNF144A (B), VRK2 (C), and G3BP1 phosphorylation (D) in stress-induced U2OS cells. The stress was induced with SA (50μM) for indicated times (8~12 hours). HSP70 was used as the marker for SA-induced stress. The phosphorylation of G3BP1 is normalized to total G3BP1 and GAPDH was used as loading control (n=3). (**E-F**) Representative immunoblot of RNF144A, VRK2, and G3BP1 phosphorylation in stress-induced A549 cells (E) and MDA-MB 231 cells (F). The stress was induced with SA (50μM) for 12 hours. P53 was used as the marker for SA-induced stress. The phosphorylation of G3BP1 is normalized to total G3BP1 and GAPDH was used as loading control. (**G**) Representative immunoblot of stress-induced U2OS cells. The stress was induced with cisplatin (50μM) for indicated times (8~12 hours). p53 was used as the marker for cisplatin-induced stress. (**H-I**) The cell viability of VRK2-deficient A549 and MDA-MB-231 cells under SA-mediated stress was measured through CCK-8 assay. The stress was induced with 50μM of SA for 24 hours or 48 hours (n=8). n.s., not significant, * *p* ≤ 0.05, ** *p* ≤ 0.01; ordinary one-way ANOVA with Tukey’s multiple comparison test was performed for (**B,C,D**). The “n” represents the number of independent experiments. Error bars indicate SDs.


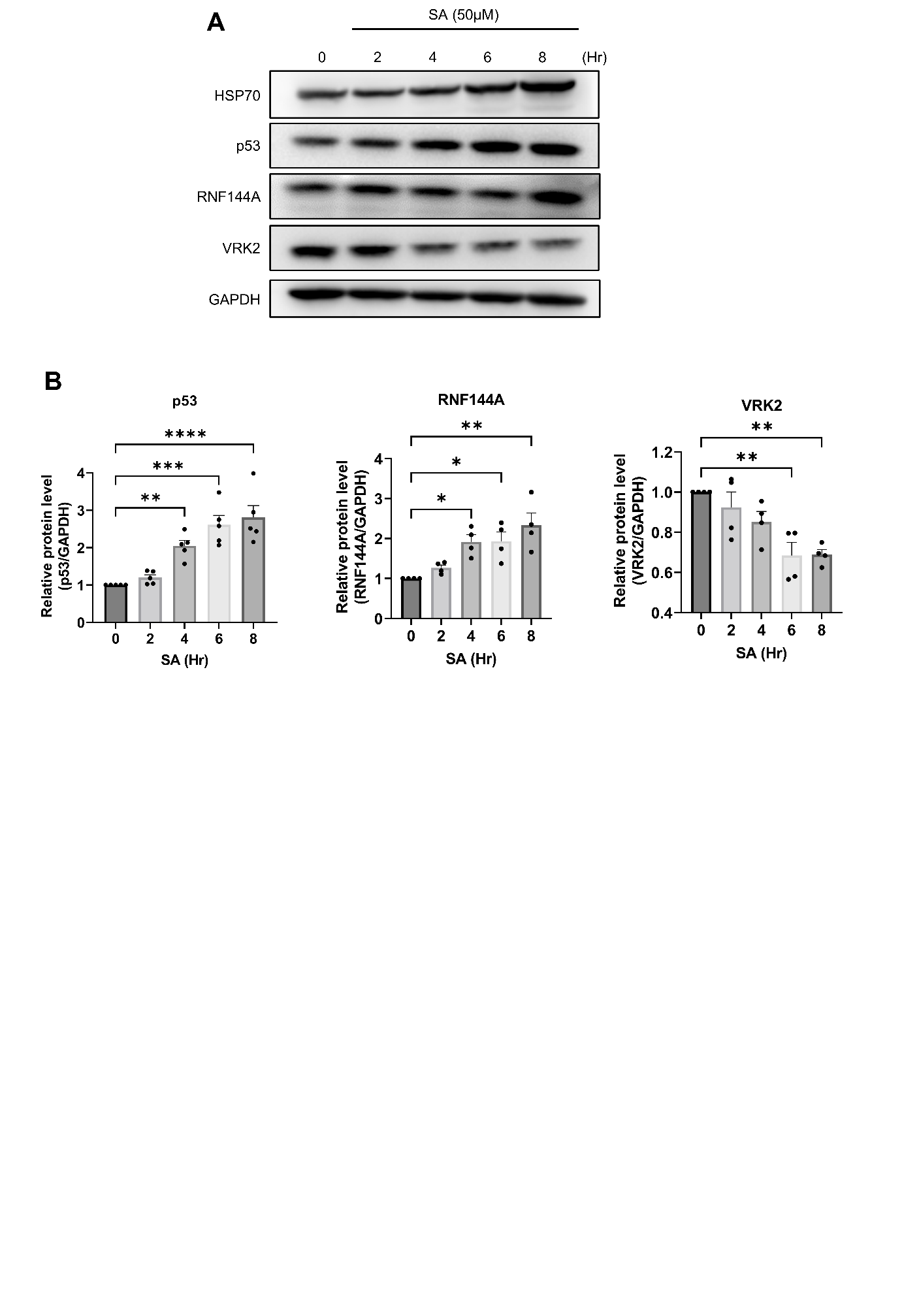


**Fig. S5. Increased level of p53 under SA-induced stress.** (**A-B**) Representative immunoblot (A) and the quantification (B) of p53, RNF144A, and VRK2 in stress-induced U2OS cells. The stress was induced with SA (50μM) for indicated times (2~8 hours). HSP70 was used as the marker for SA-induced stress. GAPDH was used for normalization (n=3). * *p* ≤ 0.05, ** *p* ≤ 0.01, *** *p* ≤ 0.001, **** *p* ≤ 0.0001; ordinary one-way ANOVA with Tukey’s multiple comparison test was performed for (**B**). The “n” represents the number of independent experiments. Error bars indicate SDs.


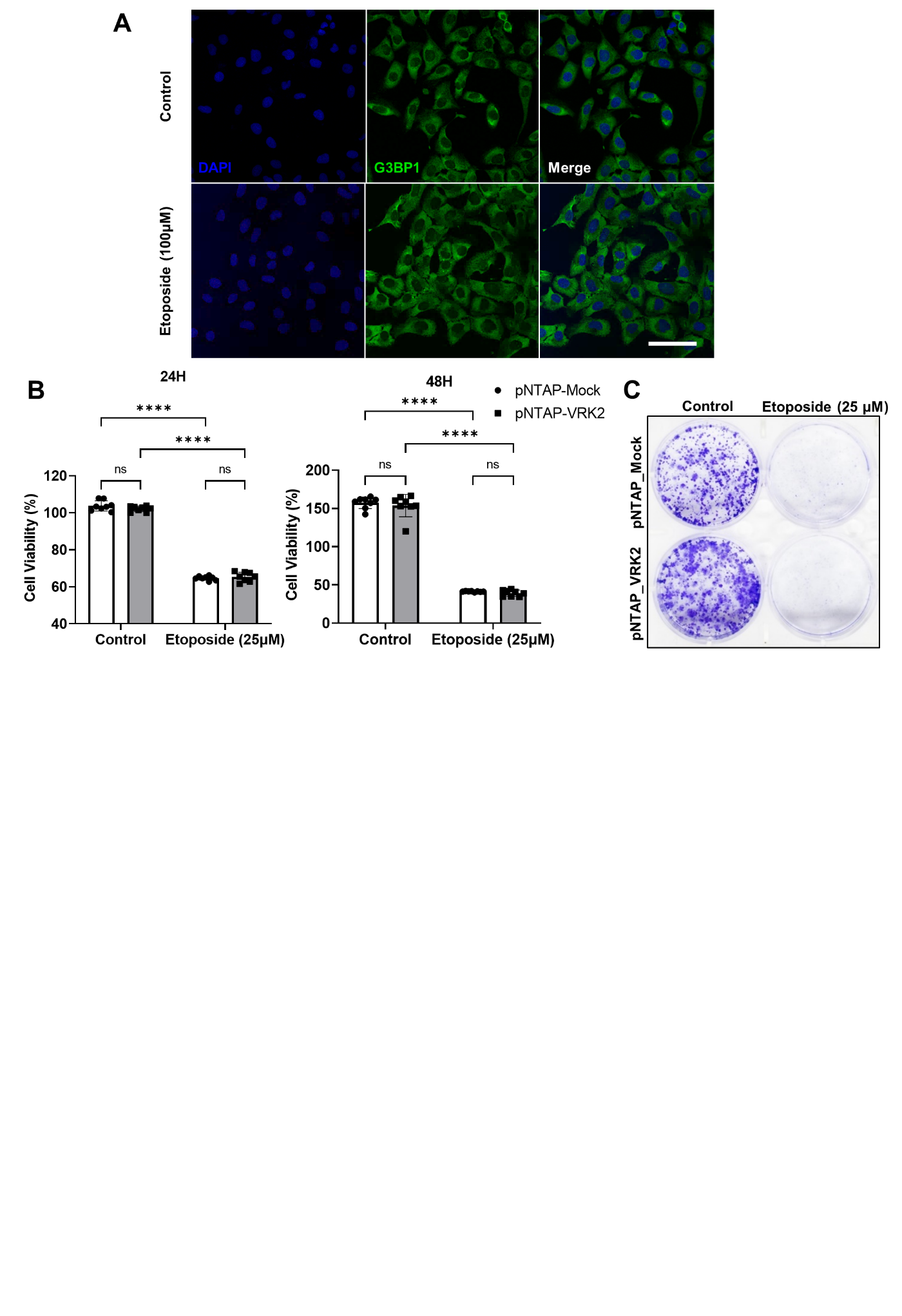


**Fig. S6. RNF-VRK2-G3BP1 axis does not participate in etoposide-mediated stress.** (**A**) Representative image of SG formation in U2OS cells. The stress was induced by the treatment with etoposide (100μM, 2 hours). SGs were stained with G3BP1 (green) and the nuclei of cells were stained with Hoechst 33342 (blue). Scale bar = 50µm. (**B**) The cell viability of pNTAP-VRK2 stably expressing U2OS cells were measured through CCK-8 assay. The stress was induced with 25μM of etoposide for 24 hours or 48 hours (n=8). (**C**) Representative image of colony formation assay performed with pNTAP-VRK2 stably expressing U2OS cells. pNTAP-VRK2 stably expressing U2OS cells were treated with etoposide (25μM) from 4^th^ day to 7^th^ day. n.s., not significant, **** *p* ≤ 0.0001; two-way ANOVA with Tukey’s multiple comparison test was performed for (**B**). The “n” represents the number of independent experiments. Error bars indicate SDs.


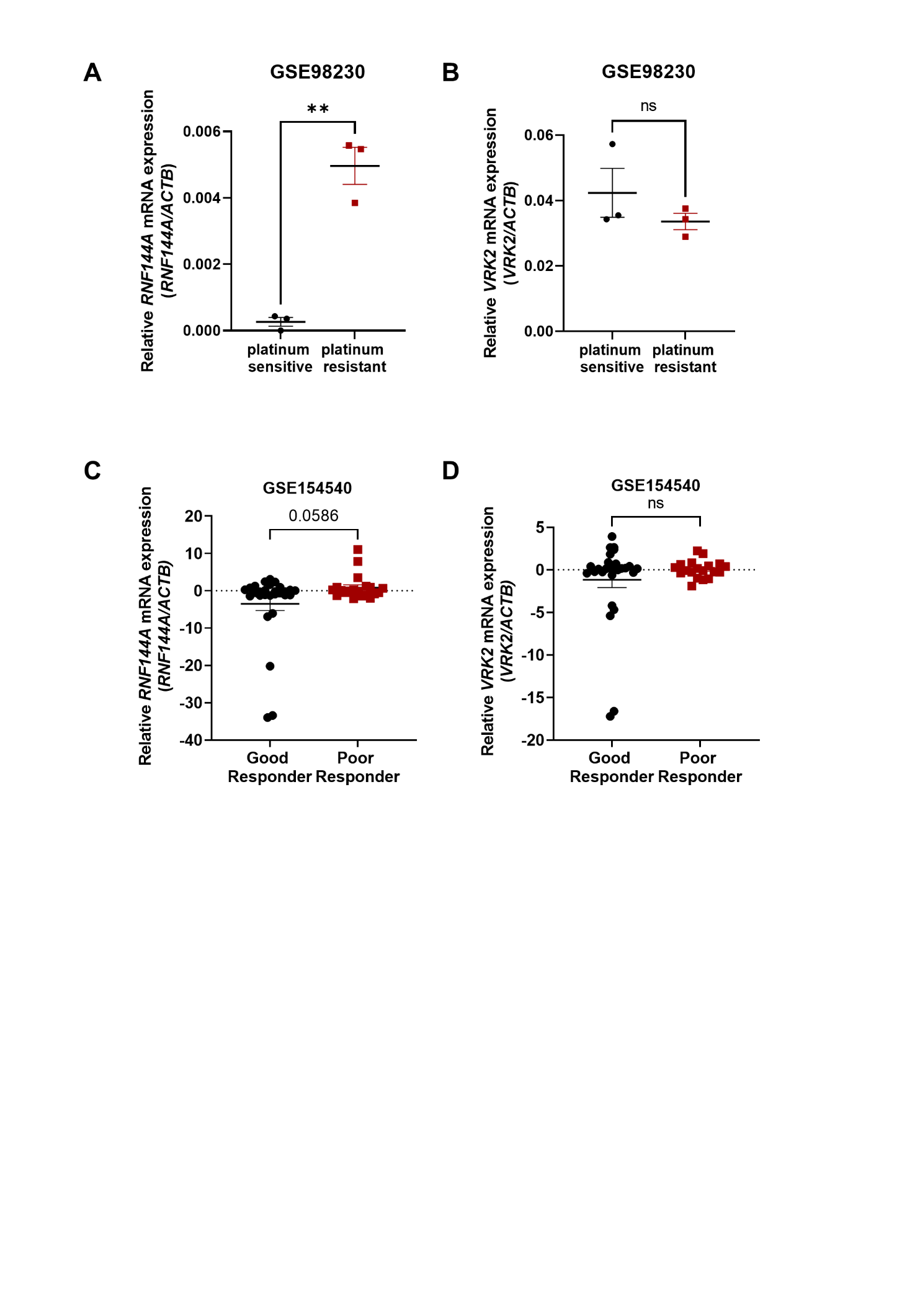


**Fig. S7. Correlation between the level of RNF144A-VRK2-G3BP1 and chemotherapy resistant patient.** (**A,B**) Analysis of publicly available gene data set from GSE98230. The mRNA expression of *RNF144A* (A) and *VRK2* (B) was compared between platinum-sensitive A2780 ovarian cancer cells and platinum-resistant A2780 ovarian cancer cell. mRNA level of *ACTB* was used for normalization (n=3). (**C,D**) Analysis of publicly available gene data set from GSE154540. The mRNA expression of *RNF144A* (C) and *VRK2* (D) was compared between cisplatin-sensitive osteosarcoma patients and cisplatin-resistant osteosarcoma patients. mRNA level of *ACTB* was used for normalization (n=29 for cisplatin-sensitive patients and n=21 for cisplatin-resistant patients). n.s., not significant, ** *p* ≤ 0.01, **** *p* ≤ 0.0001; unpaired Student’s t test was performed for (**A**,**B**,**C,D**). The “n” represents the number of independent experiments. Error bars indicate SDs.
