# Supplementary material for: RNF144A-VRK2-G3BP1 axis regulates stress granule assembly
Source: Cell Death Discov. 2025 Apr 9;11:158. doi: 10.1038/s41420-025-02460-6 (PMC11982375; doi:10.1038/s41420-025-02460-6)
Supplement: Supplementary file 2 — Original Data [file 41420_2025_2460_MOESM2_ESM.docx]

Supplementary Material for

**RNF144A-VRK2-G3BP1 axis regulates stress granule assembly**

Sung Wook Kim^±^, Jae Lee^±^, Kyung Won Jo, Young-Hun Jeong, Won Sik Shin, and Kyong-Tai Kim*

^±^These authors contributed equally to this work.

*Corresponding authors E-mail: [ktk@postech.ac.kr](mailto:ktk@postech.ac.kr)

**This file includes:**

Western Blot Raw Data

**Western Blot Raw data.**

[Figure 1A]

G3BP1

pNTAP-VRK2

55 kD

VRK2

55 kD

GAPDH

40 kD

[Figure 1B]

VRK2

55 kD

GST-G3BP1

GST

70 kD

[Figure 1D]

VRK2

55 kD

p-G3BP1(S149)

55 kD

G3BP1

55 kD

GAPDH

40 kD

[Figure 1F]

Flag

p-G3BP1(S149)

G3BP1

GAPDH

55 kD

55 kD

55 kD

40 kD

[Figure 3A]

HSP70

VRK2

Phospho-G3BP1

(S149)

G3BP1

GAPDH

70 kD

55 kD

55 kD

55 kD

40 kD

[Figure 3D]

HSP70

70 kD

[Figure 4B #1]

RNF144A

VRK2

GAPDH

40 kD

55 kD

40 kD

[Figure 4B #2]

RNF144A

VRK2

GAPDH

40 kD

55 kD

40 kD

[Figure 4D]

RNF144A

VRK2

GAPDH

40 kD

55 kD

40 kD

[Figure 4G #1]

RNF144A

VRK2

GAPDH

40 kD

55 kD

40 kD

[Figure 4G #2]

RNF144A

VRK2

GAPDH

40 kD

55 kD

40 kD

[Figure 4G #3]

RNF144A

VRK2

GAPDH

40 kD

55 kD

40 kD

[Figure 4J #1]

RNF144A

pNTAP-VRK2

GAPDH

VRK2

40 kD

55 kD

40 kD

[Figure 4J #2]

RNF144A

pNTAP-VRK2

GAPDH

VRK2

40 kD

55 kD

40 kD

[Figure 4J #3]

RNF144A

pNTAP-VRK2

GAPDH

VRK2

40 kD

55 kD

40 kD

[Figure 4K]

VRK2

GST-RNF144A

GST

55 kD

55 kD

[Figure 4L]

Ubiquitin

VRK2

(Streptavidin)

VRK2

(Input)

RNF144A

GAPDH

[Figure 5A #1]

HSP70

RNF144A

VRK2

p-G3BP1

(S149)

G3BP1

GAPDH

40 kD

70 kD

55 kD

55 kD

55 kD

40 kD

[Figure 5A #2]

HSP70

RNF144A

VRK2

p-G3BP1

(S149)

G3BP1

GAPDH

70 kD

40 kD

55 kD

55 kD

55 kD

40 kD

[Figure 5D #1]

HSP70

RNF144A

VRK2

p-G3BP1

(S149)

G3BP1

GAPDH

70 kD

40 kD

55 kD

55 kD

55 kD

40 kD

[Figure 5D #2]

HSP70

RNF144A

VRK2

p-G3BP1

(S149)

G3BP1

GAPDH

70 kD

40 kD

55 kD

55 kD

55 kD

40 kD

[Figure 5D #3]

HSP70

RNF144A

VRK2

p-G3BP1

(S149)

G3BP1

GAPDH

70 kD

40 kD

55 kD

55 kD

55 kD

40 kD

[Figure 6C]

p53

RNF144A

VRK2

p-G3BP1

(S149)

G3BP1

GAPDH

55 kD

40 kD

55 kD

55 kD

55 kD

40 kD

[Supplementary Figure 3B #1]

RNF144A

VRK2

GAPDH

55 kD

40 kD

40 kD

[Supplementary Figure 3B #2]

RNF144A

VRK2

GAPDH

55 kD

40 kD

40 kD

[Supplementary Figure 4A #1]

RNF144A

VRK2

GAPDH

HSP70

Phospho-G3BP1 (S149)

G3BP1

70 kD

40 kD

55 kD

55 kD

55 kD

40 kD

[Supplementary Figure 4A #2]

RNF144A

VRK2

GAPDH

HSP70

Phospho-G3BP1 (S149)

G3BP1

70 kD

40 kD

55 kD

55 kD

55 kD

40 kD

[Supplementary Figure 4A #3]

RNF144A

VRK2

GAPDH

HSP70

Phospho-G3BP1 (S149)

G3BP1

70 kD

40 kD

55 kD

55 kD

55 kD

40 kD

[Supplementary Figure 4E]

RNF144A

VRK2

GAPDH

p53

Phospho-G3BP1 (S149)

G3BP1

55 kD

40 kD

55 kD

55 kD

55 kD

40 kD

[Supplementary Figure 4F]

RNF144A

VRK2

GAPDH

p53

Phospho-G3BP1 (S149)

G3BP1

55 kD

40 kD

55 kD

55 kD

55 kD

40 kD

[Supplementary Figure 4G]

RNF144A

VRK2

GAPDH

p53

Phospho-G3BP1 (S149)

G3BP1

55 kD

40 kD

55 kD

55 kD

55 kD

40 kD

[Supplementary Figure 5A #1]

RNF144A

VRK2

GAPDH

p53

HSP70

70 kD

55 kD

40 kD

55 kD

40 kD

[Supplementary Figure 5A #2]

RNF144A

VRK2

GAPDH

p53

HSP70

70 kD

55 kD

40 kD

55 kD

40 kD

[Supplementary Figure 5A #3]

RNF144A

VRK2

GAPDH

p53

HSP70

70 kD

55 kD

40 kD

55 kD

40 kD
